# Multiply robust estimator for the difference in survival functions using pseudo-observations

**DOI:** 10.1186/s12874-023-02065-6

**Published:** 2023-10-23

**Authors:** Ce Wang, Kecheng Wei, Chen Huang, Yongfu Yu, Guoyou Qin

**Affiliations:** 1 Department of Biostatistics, Key Laboratory for Health Technology Assessment, National Commission of Health, Key Laboratory of Public Health Safety of Ministry of Education, School of Public Health, Fudan University, Shanghai, China; 2 Shanghai Institute of Infectious Disease and Biosecurity, Shanghai, China

**Keywords:** Survival function, Survival outcome, Empirical likelihood, Propensity score, Multiply robust

## Abstract

**Background:**

When estimating the causal effect on survival outcomes in observational studies, it is necessary to adjust confounding factors due to unbalanced covariates between treatment and control groups. There is no study on multiple robust method for estimating the difference in survival functions. In this study, we propose a multiply robust (MR) estimator, allowing multiple propensity score models and outcome regression models, to provide multiple protection.

**Method:**

Based on the previous MR estimator (Han 2014) and pseudo-observation approach, we proposed a new MR estimator for estimating the difference in survival functions. The proposed MR estimator based on the pseudo-observation approach has several advantages. First, the proposed estimator has a small bias when any PS and OR models were correctly specified. Second, the proposed estimator considers the advantage pf the pseudo-observation approach, which avoids proportional hazards assumption. A Monte Carlo simulation study was performed to evaluate the performance of the proposed estimator. And the proposed estimator was used to estimate the effect of chemotherapy on triple-negative breast cancer (TNBC) in real data.

**Results:**

The simulation studies showed that the bias of the proposed estimator was small, and the coverage rate was close to 95% when any model for propensity score or outcome regression is correctly specified regardless of whether the proportional hazard assumption holds, finite sample size and censoring rate. And the simulation results also showed that even though the propensity score models are misspecified, the bias of the proposed estimator was still small when there is a correct model in candidate outcome regression models. And we applied the proposed estimator in real data, finding that chemotherapy could improve the prognosis of TNBC.

**Conclusions:**

The proposed estimator, allowing multiple propensity score and outcome regression models, provides multiple protection for estimating the difference in survival functions.

The proposed estimator provided a new choice when researchers have a "difficult time" choosing only one model for their studies.

**Supplementary Information:**

The online version contains supplementary material available at 10.1186/s12874-023-02065-6.

## Background

Inferring the causal effect between specific treatment (exposure or intervention) and the outcome is the primary goal of much-applied research. Survival outcomes are common in epidemiologic studies and require special handling due to being subject to the right censoring during follow-up. In randomized controlled trials, the difference in survival functions based on Kaplan–Meier (KM) estimator between treatment and control groups can be used to assess the causal effect of treatment on survival outcomes [1, 2]. However, when treatment assignment is not randomized in observational studies, there are some unbalanced covariates between treatment and control groups, which leads to a biased estimation of the difference in survival functions [3]. There are two general approaches to eliminating or reducing confounding biases. The first approach is direct confounding adjustment via an outcome regression (OR) model such as Cox model [4]. The survival function in the control or treatment group is estimated by averaging the fitted values from the outcome regression model for each group, but the estimation may be biased when the proportional hazards assumption fails. Another approach is combing KM estimator with inverse probability weighting (IPW), where each subject is weighted by the inverse of the probability of receiving the treatment, known as propensity score (PS), thereby balancing the distribution of covariates between control and treatment groups [5, 6]. IPW method relies on the PS model reflecting the relationship of confounding factors with treatment [7]. The two methods above are unbiased only if the OR and PS models are correctly specified, which are usually unknown in practice.

There is growing interest in developing doubly robust (DR) methods for estimating the average causal effect (ACE) of survival outcomes to protect against potential misspecification of OR or PS model [4, 8–10]. DR methods are based on combing the IPW and direct confounding adjustment methods and provide double protection from model misspecification, as DR methods are valid when either one of the OR or PS models is specified correctly. However, DR methods do not provide sufficient protection for estimation consistency, as they allow only one model for IPW and one for direct confounding adjustment. Consistency of DR methods is still lost when PS and OR models are both misspecified [11, 12].

Compared to DR methods, multiply robust approaches allow multiple PS or OR models, increasing the likelihood of including the correct model. And multiply robust approaches are consistent when the candidate models contain correctly specified models [13]. Although multiply robust approaches are used for casual inference [14] or estimating a population mean with missing values [11, 12], they are proposed in the context of a non-survival outcome. Recently, Shu et al. [13] proposed a multiply robust approach for estimating marginal hazard ratio, yet the method only includes PS models, not OR models.

In this study, we proposed a multiply robust (MR) estimator, including multiple PS and OR models, for estimating the difference in survival functions, which has not been studied in the literature.

## Methods

### Notation

For each subject $i(i=\mathrm{1,2},\dots ,n$), we observe a $p$-dimensional vector of covariates ${{\varvec{X}}}_{i}$, treatment status ${Z}_{i}$ (i.e., it could be treatment status: ${Z}_{i}=1$ if treated and ${Z}_{i}=0$ if untreated), observed survival time ${T}_{i}$ (the smaller value of true survival time ${\widetilde{T}}_{i}$ and censoring time ${C}_{i}$) and censoring indicator ${\delta }_{i}({\delta }_{i}=1$ when ${T}_{i}={\widetilde{T}}_{i}$ and ${\delta }_{i}=0$ when ${T}_{i}={C}_{i}$). We assume that true survival time ${\widetilde{T}}_{i}$ and censoring time are independent, given ${{\varvec{X}}}_{i}$ and ${Z}_{i}$.

Note that $\left({T}^{1},{T}^{0}\right)$ represent a pair of potential survival times and cannot be observed simultaneously for a subject, where ${T}^{1}$ represents the one that would occur if every subject were treated and ${T}^{0}$ represents the one that would happen if every subject were untreated. We relate the observed survival time to the potential survival times according to the consistency assumption, that is, $T={T}^{Z}=Z{T}^{1}+\left(1-Z\right){T}^{0}$.The difference in survival functions between treatment and control groups is defined to be

1 $$\Delta \left(t\right)=E\left[\mathrm{I}\left({T}^{1}>t\right)\right]-E\left[\mathrm{I}\left({T}^{0}>t\right)\right]={S}^{1}\left(t\right)-{S}^{0}\left(t\right)$$

where $t$ is the time point at which the difference in survival functions is estimated. where ${S}^{1}\left(t\right)$ is the survival function of the treatment group and ${S}^{0}\left(t\right)$ is the survival function of the control group.

### Pseudo-observations for survival outcomes

Although survival functions for every individual can not be observed, they can be estimated by the pseudo-observation method proposed by Andersen et al. [15] and used to estimate the survival functions at a single time point [16]. Further, this approach was applied to estimate ACE of survival outcomes without considering assumptions of proportional hazards [17]. The pseudo-observation for $ith$ individual survival function at a fixed time point $t$ is defined as

2 $${\widehat{S}}_{i}\left(t\right)=n\widehat{S}\left(t\right)-\left(n-1\right){\widehat{S}}^{-i}\left(t\right)$$

where $\widehat{S}\left(t\right)$ and ${\widehat{S}}^{-i}\left(t\right)$ are the KM estimator using all samples and leaving out $ith$ individual, respectively. Graw et al. [18] have examined the asymptotic properties of the pseudo observations for competing risk models, also applying to ${\widehat{S}}_{i}\left(t\right)$.

### Pseudo-observation-based IPW

In observational studies, direct comparison of survival functions between two groups may lead to a biased causal effect estimation due to unbalanced covariates between two groups. The IPW method based on the PS $\pi \left({\varvec{X}}\right)$ is commonly used to adjust for confounding bias. We assume that there is no unmeasured confounding and positivity $(0<\pi \left({\varvec{X}}\right)<1)$ [3]. The ACE can be estimated by IPW estimator in the context of a non-survival outcome as follows

3 $${\widehat{\Delta }}_{ipw}=\frac{1}{n}\sum_{i=1}^{n}\left[\frac{{Z}_{i}{Y}_{i}}{{\widehat{\pi }}_{i}}-\frac{{(1-Z}_{i}){Y}_{i}}{1-{\widehat{\pi }}_{i}}\right]$$

where ${Y}_{i}$ is the *i*th individual outcome (continuous or discrete outcomes), and $\widehat{\pi }$ is the estimated values of $\pi \left({\varvec{X}}\right)$, for example, derived from the logistic regression models.

For time-to-event outcomes, adapting (3) for estimating $\Delta \left(t\right)$ is difficult as we do not observe individual survival functions; yet, the individual survival functions can be obtained according to (2). Once the individual survival functions are obtained, it is easy to estimate the $\Delta \left(t\right)$ for a fixed time point $t$ with (3), and the IPW estimator based on the pseudo-observation method is defined as [4, 17],

4 $${\widehat{\Delta }\left(t\right)}_{ipw}=\frac{1}{n}\sum_{i=1}^{n}\left[\frac{{Z}_{i}{\widehat{S}}_{i}\left(t\right)}{{\widehat{\pi }}_{i}}-\frac{{(1-Z}_{i}){\widehat{S}}_{i}\left(t\right)}{1-{\widehat{\pi }}_{i}}\right]$$

### Pseudo-observation-based direct confounding adjustment method

The direct confounding adjustment method for eliminating confounding bias in an OR model is another method to estimate ACE in observational studies. For time-to-event outcomes, Cox model is the most commonly used to be as an outcome regression model

5 $$h\left(t|{{\varvec{X}}}_{i},{Z}_{i}\right)={h}_{0}\left(t\right)\mathrm{exp}\left({{\varvec{\beta}}}^{{\varvec{T}}}{{\varvec{X}}}_{i}+\gamma {Z}_{i}\right)$$

where ${h}_{0}\left(t\right)$ is the baseline hazard function, ${\varvec{\beta}}$ is a set of parameters of covariates and $\gamma$ is the parameter of treatment variable $Z$. According to (5), we can estimate individual survival function $S\left(t|{{\varvec{X}}}_{i},{Z}_{i}\right)$ for the $ith$ subject given ${{\varvec{X}}}_{i}$ and ${Z}_{i}$

6 $$S\left(t|{{\varvec{X}}}_{i},{Z}_{i}\right)={S}_{0}(t{)}^{\mathrm{exp}\left({{\varvec{\beta}}}^{{\varvec{T}}}{{\varvec{X}}}_{i}+\gamma {Z}_{i}\right)}$$

where ${S}_{0}\left(t\right)$ can be estimated by fitting the Cox model. The survival functions in treatment and control are estimated by averaging individual survival functions estimated from (6), respectively. And $\Delta \left(t\right)$ can be estimated by directly comparing the survival functions between two groups. However, this method requires the proportional hazards assumption.

Pseudo observations estimated from $\left(2\right)$ can be used to predict individual survival functions with covariates and treatment variable for a fixed time point $t$, which avoids the proportional hazards assumption [4, 17]. Specifically, let ${\varvec{t}}=\left\{{t}_{1},\dots ,\left.{t}_{h}\right\}\right.$ as the set of distinct times and define the pseudo-observation for subject $i$ at time ${t}_{h}$ as ${\widehat{S}}_{i}\left({t}_{h}\right)$, where $h=1,\dots ,H$ and $i=1,\dots ,n$. Then we can convert the Cox model to a generalized linear model (GLM) with a suitable link function $\mathrm{g}\left(x\right)=\mathrm{log}\left(-\mathrm{log}\left(x\right)\right)$ [15],

7 $$\mathrm{g}\left( {S}_{i}({t}_{h})\right)={\xi }^{{t}_{h}}+\gamma {Z}_{i}+{{\varvec{\beta}}}^{{\varvec{T}}}{{\varvec{X}}}_{{\varvec{i}}}$$

Where ${\xi }^{{t}_{h}}$ is the intercept term for time ${t}_{h}$, and $\gamma$ and ${\varvec{\beta}}$ are regression parameters. Note that, although ${\xi }^{{t}_{h}}$ are time specific, $\gamma$ and ${\varvec{\beta}}$ are shared between different time points ${\varvec{t}}=\left\{{t}_{1},\dots ,\left.{t}_{h}\right\}\right.$*.* Estimating the unknown parameters $\gamma$, ${\varvec{\beta}}$ and ${{\varvec{\beta}}}_{{\varvec{H}}}=\left\{{\xi }^{1},\dots ,\left.{\xi }^{{t}_{h}}\right\}\right.$ could be solved by the following generalized estimating equation (GEE)

8 $$\sum_{i=1}^{n}\frac{\partial {\mathrm{g}}^{-1}\left({\varvec{t}},{{\varvec{\beta}}}^{*};{Z}_{i},{{\varvec{X}}}_{{\varvec{i}}}\right)}{\partial \beta }{V}_{i}^{-1}\left\{{\widehat{S}}_{i}\left({\varvec{t}}\right)-{\mathrm{g}}^{-1}\left({\varvec{t}},{{\varvec{\beta}}}^{*};{Z}_{i},{{\varvec{X}}}_{{\varvec{i}}}\right)\right\}=U\left({{\varvec{\beta}}}^{\boldsymbol{*}}\right)$$

where ${{\varvec{\beta}}}^{\boldsymbol{*}}=({{\varvec{\beta}}}_{{\varvec{H}}},\boldsymbol{ }\boldsymbol{ }\gamma \boldsymbol{ },{\varvec{\beta}})$**,** ${\widehat{S}}_{i}\left({\varvec{t}}\right)=({{\widehat{S}}_{i}\left({t}_{1}\right),\dots ,{\widehat{S}}_{i}\left({t}_{h}\right))}^{T}$ and ${V}_{i}^{-1}$ is a working covariance matrix which may account for the correlation structure inherent to the pseudo-observations [17]. The difference in survival functions with direct confounding adjustment based on the pseudo-observation method under model (7) can be estimated as

9 $${\widehat{\Delta }\left(t\right)}_{OR}=\frac{1}{n}\sum_{i=1}^{n}\left\{{\mathrm{g}}^{-1}\left({\widehat{\xi }}^{t}+\widehat{\gamma }+{\widehat{{\varvec{\beta}}}}^{{\varvec{T}}}{{\varvec{X}}}_{{\varvec{i}}}\right)-{\mathrm{g}}^{-1}\left({\widehat{\xi }}^{t}+{\widehat{{\varvec{\beta}}}}^{{\varvec{T}}}{{\varvec{X}}}_{{\varvec{i}}}\right)\right\}$$

If the outcome regression model (5) is correctly specified, the ${\widehat{\Delta }\left(t\right)}_{OR}$ is consistent.

## Multiply robust estimator for the difference in survival functions

We combined the advantages of previous MR (Han, 2014) and PO method to proposed the proposed MR method. The proposed method could consider a set of OR models and a sets of PS models and also avoid the proportional hazards assumption. The step of estimating the difference in survival functions is similar to the previous MR (Han, 2014). First, specifying multiple PS models and multiple OR models. Second, estimating weights of individual in the treatment and control groups, respectively. Finally, estimating the difference in survival functions between treatment and control groups.

Specifically, to achieve multiple robustness, we specify multiple PS models and multiple OR models, $\mathcal{P}=\left\{{\pi }^{l}\left({\varvec{X}}\right),l=1, 2, 3,\dots ,L\right\}$ for the propensity score and $\mathcal{M}=\left\{{m}_{Z}^{k}\left(X\right),k=1, 2, 3,\dots ,K\right\}$ for outcome regression, where ${m}_{Z}^{k}\left({\varvec{X}}\right)={m}^{k}\left({\varvec{X}},Z\right)$. Without loss of generality, let ${\mathbb{I}}=1,\dots ,{n}_{1}$ represent the indexes for treated subjects and ${\mathbb{J}}=1,\dots ,{n}_{0}$ the indexes for untreated subjects. ${n}_{1}$ and ${n}_{0}=n-{n}_{1}$ are the size of treatment and control groups, respectively.

Second, to recover survival function from treated subjects for a time point $t$, we impose ${w}_{i}\left(i\in {\mathbb{I}}\right)$ as the weights of individual pseudo-observation ${S}_{i}\left(t\right)$ from the treated subjects, which can be estimated by maximizing $\prod_{i\in {\mathbb{I}}}{w}_{i}$ according to the following constraints:

10 $${w}_{i}\ge 0 \left(i\in {\mathbb{I}}\right)$$

11 $$\sum_{i\in {\mathbb{I}}}{w}_{i}=1$$

12 $$\sum_{i\in {\mathbb{I}}}{w}_{i}{\widehat{\pi }}^{l}\left({{\varvec{X}}}_{i}\right)={\widehat{\theta }}_{1}^{l}\left(l=1, 2, 3,\dots ,L\right)$$

13 $$\sum_{i\in {\mathbb{I}}}{w}_{i}{\widehat{m}}_{1}^{k}\left({{\varvec{X}}}_{i}\right)={\widehat{\eta }}_{1}^{k}\left(k=1, 2, 3,\dots ,K\right)$$

where ${\widehat{\theta }}_{1}^{l}={n}^{-1}{\sum }_{i=1}^{n}{\pi }^{l}\left({{\varvec{X}}}_{i}\right)$ and ${\widehat{\eta }}_{1}^{k}={n}^{-1}{\sum }_{i=1}^{n}{m}_{1}^{k}\left({{\varvec{X}}}_{i}\right)$. Similarly, we also impose ${w}_{j}\left(j\in {\mathbb{J}}\right)$ as the weights of individual pseudo-observation ${S}_{j}\left(t\right)$ from the untreated subjects, which can be estimated by maximizing $\prod_{j\in {\mathbb{J}}}{w}_{j}$ subject to the following constraints:

14 $${w}_{j}\ge 0 \left(j\in {\mathbb{J}}\right)$$

15 $$\sum_{j\in {\mathbb{J}}}{w}_{j}=1$$

16 $$\sum_{j\in {\mathbb{J}}}{w}_{j}\left({1-\widehat{\pi }}^{l}\left({{\varvec{X}}}_{j}\right)\right)={\widehat{\theta }}_{0}^{l}\left(l=1, 2, 3,\dots ,L\right)$$

17 $$\sum_{j\in {\mathbb{J}}}{w}_{j}{\widehat{m}}_{0}^{k}\left({{\varvec{X}}}_{j}\right)={\widehat{\eta }}_{0}^{k}\left(k=1, 2, 3,\dots ,K\right)$$

where ${\widehat{\theta }}_{0}^{l}={n}^{-1}{\sum }_{j=1}^{n}({1-\pi }^{l}\left({{\varvec{X}}}_{j}\right))$ and ${\widehat{\eta }}_{0}^{k}={n}^{-1}{\sum }_{j=1}^{n}{m}_{0}^{k}\left({{\varvec{X}}}_{j}\right)$. According to Lagrange multiplier method [19], it is easy to show that ${w}_{i}$ and ${w}_{j}$ maximizing the $\prod_{i\in {\mathbb{I}}}{w}_{i}$ and $\prod_{j\in {\mathbb{J}}}{w}_{j}$ are given by

18 $${\widehat{w}}_{i}=\frac{1}{{n}_{1}}\frac{1}{1+{\widehat{{\varvec{\rho}}}}_{1}^{T}{\widehat{{\varvec{g}}}}_{1}\left({{\varvec{X}}}_{i}\right)} \left(i\in {\mathbb{I}}\right)$$

19 $${\widehat{w}}_{j}=\frac{1}{{n}_{0}}\frac{1}{1+{\widehat{{\varvec{\rho}}}}_{0}^{T}{\widehat{{\varvec{g}}}}_{0}\left({{\varvec{X}}}_{j}\right)} \left(j\in {\mathbb{J}}\right)$$

where

$${\widehat{{\varvec{g}}}}_{1}{\left({\varvec{X}}\right)}^{T}=\left\{{\widehat{\pi }}^{1}\left({\varvec{X}}\right)-{\widehat{\theta }}_{1}^{1},\dots ,{\widehat{\pi }}^{L}\left({\varvec{X}}\right)-{\widehat{\theta }}_{1}^{L}, {\widehat{m}}_{1}^{1}\left({\varvec{X}}\right)-{\widehat{\eta }}_{1}^{1},\dots ,{\widehat{m}}_{1}^{K}\left({\varvec{X}}\right)-{\widehat{\eta }}_{1}^{K}\right\}$$

$${\widehat{{\varvec{g}}}}_{0}{\left({\varvec{X}}\right)}^{T}=\left\{{(1-\widehat{\pi }}^{1}\left({\varvec{X}}\right)\right)-{\widehat{\theta }}_{0}^{1},\dots ,(1-{\widehat{\pi }}^{L}\left({\varvec{X}}\right))-{\widehat{\theta }}_{0}^{L}, {\widehat{m}}_{0}^{1}\left({\varvec{X}}\right)-{\widehat{\eta }}_{0}^{1},\dots ,{\widehat{m}}_{0}^{K}\left({\varvec{X}}\right)-{\widehat{\eta }}_{0}^{K}\}$$

and ${\widehat{\rho }}_{t}^{T}=\left({\widehat{\rho }}_{t1},{\widehat{\rho }}_{t2},{\dots ,\widehat{\rho }}_{tS}\right)$ is $S=J+K$-dimensional vector satisfying the equations

20 $$\frac{1}{{n}_{1}}\sum_{i\in {\mathbb{I}}}\frac{{\widehat{{\varvec{g}}}}_{1}\left({{\varvec{X}}}_{{\varvec{i}}}\right)}{1+{\widehat{{\varvec{\rho}}}}_{1}^{T}{\widehat{{\varvec{g}}}}_{1}\left({{\varvec{X}}}_{{\varvec{i}}}\right)}=0$$

21 $$\frac{1}{{n}_{0}}\sum_{j\in {\mathbb{J}}}\frac{{\widehat{{\varvec{g}}}}_{0}\left({{\varvec{X}}}_{j}\right)}{1+{\widehat{{\varvec{\rho}}}}_{0}^{T}{\widehat{{\varvec{g}}}}_{0}\left({{\varvec{X}}}_{j}\right)}=0$$

Due to the non-negativity of ${w}_{i}$ and ${w}_{j}$, ${\widehat{\rho }}_{t}^{T}$ must satisfy that $1+{\widehat{{\varvec{\rho}}}}_{1}^{T}{\widehat{{\varvec{g}}}}_{1}\left({{\varvec{X}}}_{i}\right)>0$ and $1+{\widehat{{\varvec{\rho}}}}_{0}^{T}{\widehat{{\varvec{g}}}}_{0}\left({{\varvec{X}}}_{j}\right)>0$. The estimation of ${\widehat{w}}_{i}$ and ${\widehat{w}}_{j}$ can be solved by the Newton–Raphson algorithm [19].

Finally, the MR estimator of the difference in survival functions between treatment and control groups for the fixed time $t$ is defined as

22 $${\widehat{\Delta }\left(t\right)}_{mr}=\sum_{i\in {\mathbb{I}}}{\widehat{w}}_{i}{\widehat{S}}_{i}\left(t\right)-\sum_{j\in {\mathbb{J}}}{\widehat{w}}_{j}{\widehat{S}}_{j}\left(t\right)$$

${\widehat{S}}_{i}\left(t\right)$ and ${\widehat{S}}_{j}\left(t\right)$ are the individual survival functions estimated with PO method.

## Bootstrap confidence interval

We perform the bootstrap method to estimate the confidence interval of ${\widehat{\Delta }\left(t\right)}_{mr}$. Specifically, $n$ subjects are resampled from the original data with the replacement for $B$ times to obtain $B$ bootstrap samples, where $B$ is a user-specified number. For $b=1,\dots ,B$, let ${\widehat{\Delta }}_{mr}^{b}\left(t\right)$ be the estimated difference of survival functions from the $b$-th bootstrap sample. Then the bootstrap variance estimator for ${\widehat{\Delta }}_{mr}^{b}\left(t\right)$ is defined as

23 $$\widehat{var}\left({\widehat{\Delta }}_{mr}^{b}\left(t\right)\right)= \frac{1}{B-1}\sum_{b=1}^{B}({\widehat{\Delta }}_{mr}^{b}\left(t\right)-\frac{1}{B}\sum_{b=1}^{B}{\widehat{\Delta }}_{mr}^{b}\left(t\right){)}^{2}$$

A normality-based 95% confidence interval for ${\Delta \left(t\right)}_{mr}$ is ${\widehat{\Delta }}_{mr}^{b}\left(t\right)\pm 1.96\sqrt{\widehat{var}\left({\widehat{\Delta }}_{mr}^{b}\left(t\right)\right)}$.

## Simulation studies

### Simulation design

We conducted comprehensive simulation studies to evaluate the performance of the proposed method. There were three covariates ${X}_{1},{X}_{2}, {X}_{3}$ distributed as standard normal with mean zero and unit variance, where $\mathrm{corr}\left({X}_{1},{X}_{3}\right)=0.2$ and ${X}_{2}$ was independent of the ${X}_{1}$ and ${X}_{3}$. The treatment indicator $Z$ was simulated from the Bernoulli distribution according to the following propensity score

$$\mathrm{logit}\left[\pi \left({\varvec{X}}\right)\right]= {(X}_{1}+{X}_{2}+{X}_{3})/3$$

The survival times were simulated from a Cox-Weibull model with ${\widetilde{T}}_{1}={\left[\frac{-\mathrm{log}\left(u\right)}{{\lambda }_{1}\mathrm{exp}\left(L-\gamma \right)}\right]}^{\frac{1}{{v}_{1}}}$ for treatment group and ${\widetilde{T}}_{0}={\left[\frac{-\mathrm{log}\left(u\right)}{{\lambda }_{0}\mathrm{exp}\left(L\right)}\right]}^{\frac{1}{{v}_{0}}}$ for control group, where $L= -3+0.5*\left({X}_{1}+{X}_{2}\right)$, $\gamma =1$, $u$ was simulated from Uniform(0,1). And we defined the proportional hazard setting via setting the shape and scale parameters [(${v}_{1}=0.005, {\lambda }_{1}=3$), (${v}_{0}=0.005, {\lambda }_{0}=3$)] in the treatment and control groups (Figure S1). An independent censoring time was simulated from an exponential distribution with rates, and we set the different rates [$\mathrm{exp}\left(-4\right)or\mathrm{exp}\left(-3\right)$] to obtain censoring rates (approximately 25% or 50%).

In this simulation, we specified two models

$${\mathbb{A}}=\left\{\begin{array}{c}logit\left[{\pi }^{1}\left({\varvec{X}};{{\varvec{\beta}}}^{1}\right)\right]=\left({X}_{1},{X}_{2},{X}_{3}\right){{\varvec{\beta}}}^{1}\\ logit\left[{\pi }^{2}\left({\varvec{X}};{{\varvec{\beta}}}^{2}\right)\right]=\left({X}_{1}^{2},{X}_{2}^{2},{X}_{3}^{2}\right){{\varvec{\beta}}}^{2}\end{array}\right\}$$

for propensity score, and two models

$${\mathbb{B}}=\left\{\begin{array}{c}{m}^{1}\left({\varvec{X}},{\varvec{Z}};{{\varvec{\gamma}}}^{1}\right)=\left(1,{X}_{1},{X}_{2},Z\right){{\varvec{\gamma}}}^{1}\\ {m}^{2}\left({\varvec{X}},{\varvec{Z}};{{\varvec{\gamma}}}^{2}\right)=\left(1,{X}_{1}^{2},{X}_{2}^{2},Z\right){{\varvec{\gamma}}}^{2}\end{array}\right\}$$

for outcome regression. According to the data-generating process, ${\pi }^{1}\left({\varvec{X}};{{\varvec{\beta}}}^{1}\right)$ and ${m}^{1}\left({\varvec{X}},Z;{{\varvec{\gamma}}}^{1}\right)$ were correctly specified, while ${\pi }^{2}\left({\varvec{X}};{{\varvec{\beta}}}^{2}\right)$ and ${m}^{2}\left({\varvec{X}},Z;{{\varvec{\gamma}}}^{2}\right)$ were incorrectly specified.

We were interested in estimating the difference in survival functions $\Delta \left({t}^{*}\right)$ at ${t}^{*}=10 and 20$. We performed IPW, direct confounding adjustment, and multiply robust (MR) methods to estimate the $\Delta \left(t\right)$. To distinguish these estimation methods, two IPW-based estimators were denoted as “IPW.model1” and “IPW.model2”; two direct-confounding-adjustment-based estimators were denoted as “OR.model1” and “OR.model2”; for the MR estimators, each estimator is denoted as “MR-0000”, where each digit of the four numbers, from left to right, indicates if ${\pi }^{1}\left({\varvec{X}};{{\varvec{\beta}}}^{1}\right)$, ${\pi }^{2}\left({\varvec{X}};{{\varvec{\beta}}}^{2}\right)$, ${m}^{1}\left({\varvec{X}},Z;{{\varvec{\gamma}}}^{1}\right)$ or ${m}^{2}\left({\varvec{X}},Z;{{\varvec{\gamma}}}^{2}\right)$ is included in the estimator (“1” means yes and “0” means no).

**Fig. 1:**
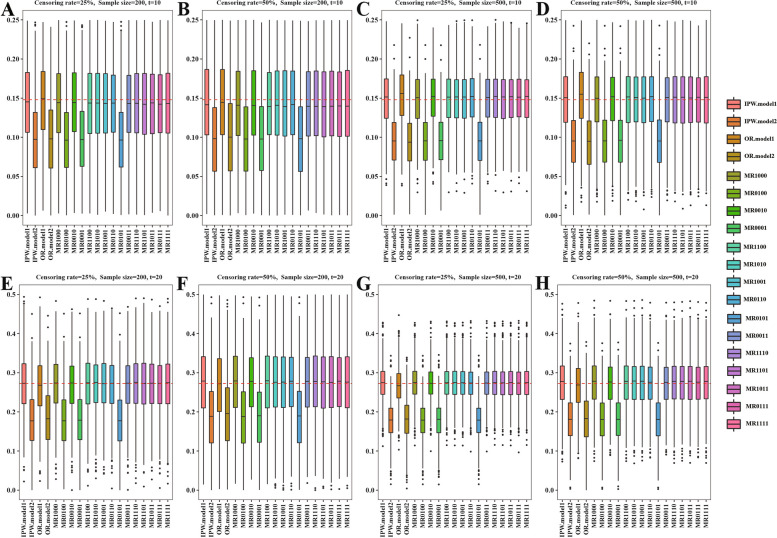
Simulation results with different sample sizes = 200 or 500 and different censoring rates = 25% or 50% in the scenario where proportional hazard assumption holds based on 1000 replication. The red dotted line represents the true value, the true values of $\Delta \left(t=10\right)$ or $\Delta \left(t=20\right)$ are 0.1480 and 0.2725, respectively. IPW: inverse probability weighting; OR: outcome regression; MR, multiply robust; MR estimators are denoted as “MR-0000”, where each digit of the four numbers, from left to right, indicates if ${\pi }^{1}\left({\varvec{X}};{{\varvec{\beta}}}^{1}\right)$, ${\pi }^{2}\left({\varvec{X}};{{\varvec{\beta}}}^{2}\right)$, ${m}^{1}\left({\varvec{X}},Z;{{\varvec{\gamma}}}^{1}\right)$ or ${m}^{2}\left({\varvec{X}},Z;{{\varvec{\gamma}}}^{2}\right)$ is included in the estimator (“1” means yes and “0” means no)

**Fig. 2:**
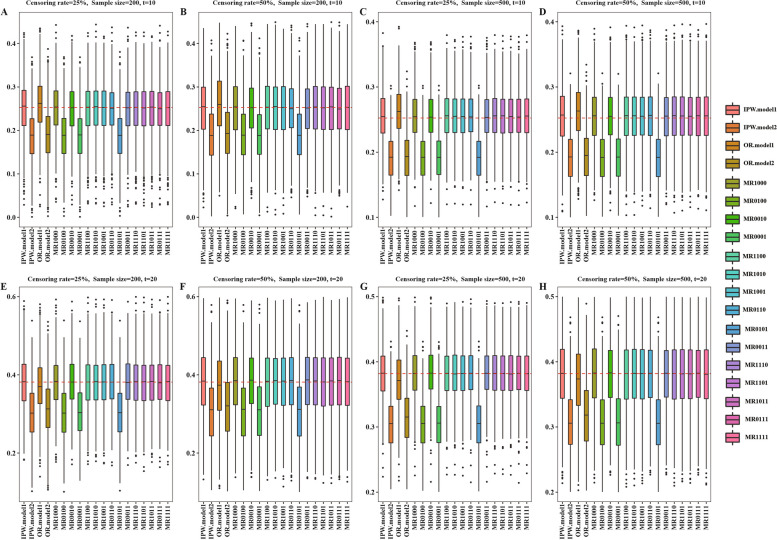
Simulation results with different sample sizes = 200 or 500 and different censoring rates = 25% or 50% in the scenario where proportional hazard assumption dose not hold based on 1000 replications. The red dotted line represents the true value, the true values of $\Delta \left(t=10\right)$ or $\Delta \left(t=20\right)$ are 0.2524 and 0.3816, respectively. IPW: inverse probability weighting; OR: outcome regression; MR, multiply robust; MR estimators are denoted as “MR-0000”, where each digit of the four numbers, from left to right, indicates if ${\pi }^{1}\left({\varvec{X}};{{\varvec{\beta}}}^{1}\right)$, ${\pi }^{2}\left({\varvec{X}};{{\varvec{\beta}}}^{2}\right)$, ${m}^{1}\left({\varvec{X}},Z;{{\varvec{\gamma}}}^{1}\right)$ or ${m}^{2}\left({\varvec{X}},Z;{{\varvec{\gamma}}}^{2}\right)$ is included in the estimator (“1” means yes and “0” means no)

**Fig. 3:**
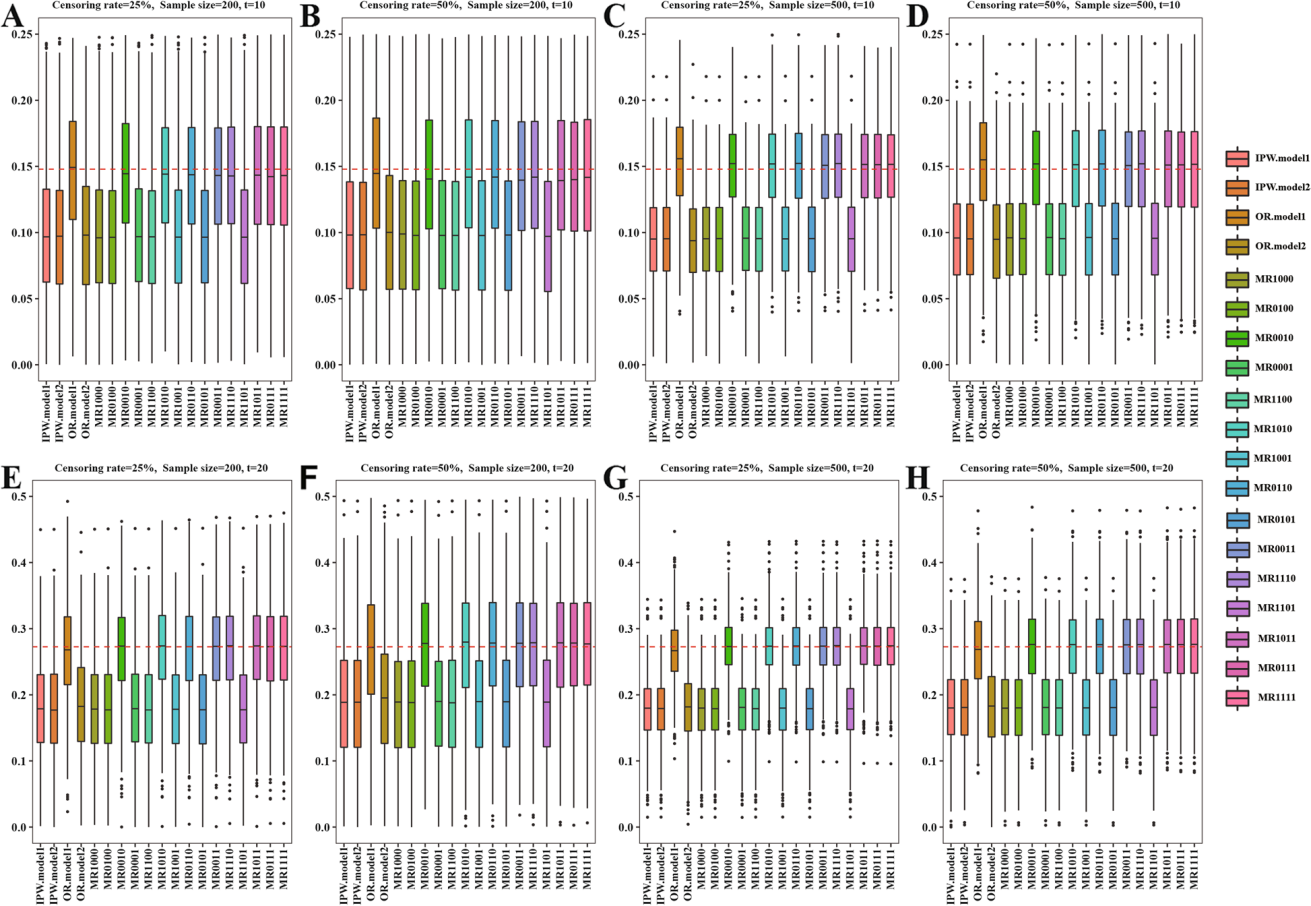
Simulation results with different sample sizes = 200 or 500 and different censoring rates = 25% or 50% in the scenario where proportional hazard assumption holds based on 1000 replications when PS models misspecified. The red dotted line represents the true value, the true values of $\Delta \left(t=10\right)$ or $\Delta \left(t=20\right)$ are 0.1480 and 0.2725, respectively. IPW: inverse probability weighting; OR: outcome regression; MR, multiply robust; MR estimators are denoted as “MR-0000”, where each digit of the four numbers, from left to right, indicates if ${\pi }^{1}\left({\varvec{X}};{{\varvec{\beta}}}^{1}\right)$, ${\pi }^{2}\left({\varvec{X}};{{\varvec{\beta}}}^{2}\right)$, ${m}^{1}\left({\varvec{X}},Z;{{\varvec{\gamma}}}^{1}\right)$ or ${m}^{2}\left({\varvec{X}},Z;{{\varvec{\gamma}}}^{2}\right)$ is included in the estimator (“1” means yes and “0” means no)

**Fig. 4:**
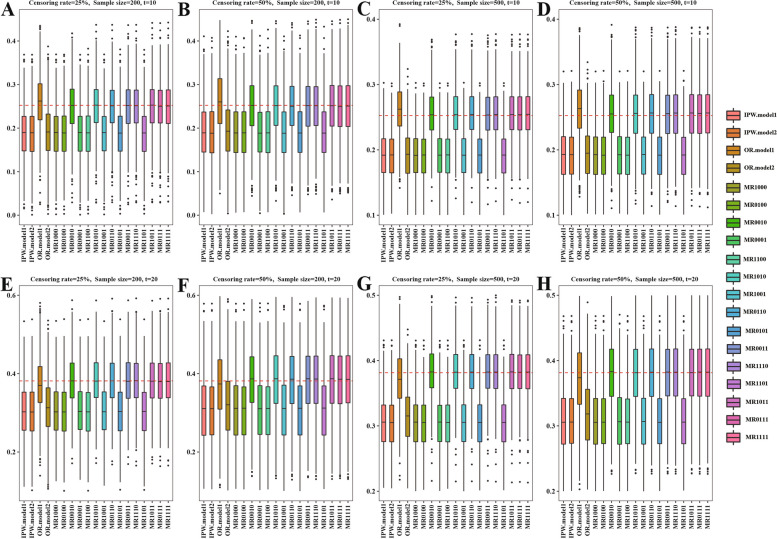
Simulation results with different sample sizes = 200 or 500 and different censoring rates = 25% or 50% in the scenario where proportional hazard assumption dose not hold based on 1000 replications when PS models misspecified. The red dotted line represents the true value, the true values of $\Delta \left(t=10\right)$ or $\Delta \left(t=20\right)$ are 0.2524 and 0.3816, respectively. IPW: inverse probability weighting; OR: outcome regression; MR, multiply robust; MR estimators are denoted as “MR-0000”, where each digit of the four numbers, from left to right, indicates if ${\pi }^{1}\left({\varvec{X}};{{\varvec{\beta}}}^{1}\right)$, ${\pi }^{2}\left({\varvec{X}};{{\varvec{\beta}}}^{2}\right)$, ${m}^{1}\left({\varvec{X}},Z;{{\varvec{\gamma}}}^{1}\right)$ or ${m}^{2}\left({\varvec{X}},Z;{{\varvec{\gamma}}}^{2}\right)$ is included in the estimator (“1” means yes and “0” means no)

In addition, we defined the non-proportional hazard setting via setting the shape scale parameters ${[(v}_{1}=0.005, {\lambda }_{1}=3), {(v}_{0}=0.005, {\lambda }_{0}=3.2)]$ in the treatment and control groups, respectively (Figure S1); and we also specified two models incorrectly

$${\mathbb{A}}=\left\{\begin{array}{cc}\mathrm{logit}[{\pi }^{1}({\varvec{X}};{{\varvec{\beta}}}^{1})]= ({X}_{1}^{2}, {X}_{2}^{2}){{\varvec{\beta}}}^{1}\\ \mathrm{logit}[{\pi }^{2}({\varvec{X}};{{\varvec{\beta}}}^{2})]=({X}_{1}^{2}, {X}_{2}^{2}, {X}_{3}^{2}){{\varvec{\beta}}}^{1}\end{array}\right\}$$

for propensity score. In the study, we investigated a sample size of $n=200$ and $n=500$ with 1000 replications. We used three evaluation criteria: mean relative bias, root mean square error (RMSE), and coverage rate. The boxplots of mean relative bias, RMSE and coverage rate were shown in the main article (Figs. 1,2, 3 and 4) and the table of estimation results were shown in the supplementary material (Table S1- S4).

### Simulation results

Figure 1 and Table S1 showed the simulation results of the difference between treatment and control groups in the proportional hazard setting with the different censoring rates (25% or 50%) and different sample sizes (200 or 500). We could draw a few conclusions from the simulation results.

1. When specifying a propensity score model $\pi \left({\varvec{X}};{\varvec{\beta}}\right)$, or an outcome regression model $m\left({\varvec{X}},Z;{\varvec{\gamma}}\right)$: the biases of IPW and MR were negligible if $\pi \left({\varvec{X}};{\varvec{\beta}}\right)$ was correctly specified (IPW.model1, MR1000). The biases of OR and MR were small if $m\left({\varvec{X}},Z;{\varvec{\gamma}}\right)$ was correctly modeled (OR.model1, MR0010), and MR0010 had the smallest RMSE among all estimators. The biases and RMSEs were large, and the coverage rates were much less than 95% if $\pi \left({\varvec{X}};{\varvec{\beta}}\right)$ or $m\left({\varvec{X}},Z;{\varvec{\gamma}}\right)$ were incorrectly specified (IPW.model2, OR.model2, MR0100, MR0001).
2. When specifying a propensity score model $\pi \left({\varvec{X}};{\varvec{\beta}}\right)$, and an outcome regression model $m\left({\varvec{X}},Z;{\varvec{\gamma}}\right)$: the biases MR were ignorable when either $\pi \left({\varvec{X}};{\varvec{\beta}}\right)$ or $m\left({\varvec{X}},Z;{\varvec{\gamma}}\right)$ was correctly specified (MR1010, MR1001, MR0110). The biases and RMSEs were severe large, and the coverage rates were well below 95% when the $\pi \left({\varvec{X}};{\varvec{\beta}}\right)$ and $m\left({\varvec{X}},Z;{\varvec{\gamma}}\right)$ were both incorrectly specified (MR0101). MR1010, MR1001 and MR0110 had almost the same RMSEs, which were smaller than IPW.model1 and OR.model1.
3. When specifying multiply propensity score models $\pi \left({\varvec{X}};{\varvec{\beta}}\right)$, and multiply outcome regression model models $m\left({\varvec{X}},{\varvec{Z}};{\varvec{\gamma}}\right)$: the small biases of MR1100, MR0011, MR1110, MR1101, MR1011, MR0111, and MR1111 well suggested the multiple robustness of our proposed methods. Based on our simulation results, the RMSEs of those estimators were almost all smaller than IPW.model1 and OR.model1, suggesting that adding more models did not lead to a significant increase in RMSE.

In addition, Fig. 2 and Table S2 showed the estimation results in the non-proportional hazard setting. Figures 3 and 4 and Tables S3 and S4 showed the simulation results in proportional and non-proportional hazard settings with two PS models specified incorrectly. Similar patterns were observed in Figs. 2, 3 and 4 and Tables S2-S4.

In conclusion, the simulation results showed that our MR method could provide more protection against model misspecification even when PS models are misspecified in the proportional or non-proportional hazard settings.

### Empirical study

To illustrate the proposed method, we analyzed the real-world data on triple-negative breast cancer (TNBC) from Surveillance, Epidemiology, and End Results (SEER) [20]. TNBC, characterized by an absence of estrogen, progesterone receptors, and human epidermal growth factor receptor 2, has high invasiveness, high metastatic potential, proneness to relapse, and poor prognosis [21]. Chemotherapy remains the primary treatment for TNBC and improves the prognosis of TNBC patients [22]. The objective of the study was to estimate the effect of chemotherapy on TNBC. Baseline information included marital status, race, laterality, grade, The American Joint Committee on Cancer (AJCC) stage, distant metastasis, age, surgery, and chemotherapy. Inclusion criteria are: (1) patients diagnosed between 2010 and 2012, (2) patients aged between 20 and 79, (3) one primary tumor only, (4) complete baseline and survival information. A total of 10,613 patients were included in the analysis, with 79.9% opting for chemotherapy and a censoring rate of 80%. The baseline information was summarized in Table 1. We found that the chemotherapy group had a higher proportion of patients with III/IV stage, $\ge 50$ years old, receiving radiotherapy and surgery compared to the non-chemotherapy group.

**Table 1:** Baseline characteristics between chemotherapy and non-chemotherapy groups

	Non-chemotherapy^a^	Chemotherapy
Survival (months)	44.61 ± 18.20	45.40 ± 16.82
Death		
Yes	463 (21.7)	1876 (22.1)
No	1673 (78.3)	6601 (77.9)
Marital Status		
Yes^b^	1190 (55.7)	5176 (61.1)
No^c^	946 (44.3)	3301 (38.9)
Laterality		
Left	1105 (51.7)	4333 (51.1)
Right	1031 (48.3)	4144 (48.9)
Race		
Black	409 (19.1)	1798 (21.2)
Non-black^d^	1727 (80.9)	6679 (78.8)
Grade		
Well/Moderately	1545 (72.3)	7302 (86.1)
Poorly/Undifferentiated	591 (27.7)	1175 (13.9)
Stage		
I/II	1926 (90.2)	6551 (77.3)
III/IV	210 (9.8)	1926 (22.7)
Distant metastasis		
Yes	101 (4.7)	727 (8.6)
No	2035 (95.3)	7750 (91.4)
Age		
$<$ 50	382 (17.9)	3054 (36.0)
$\ge$ 50	1754 (82.1)	5423 (64.0)
Radiotherapy		
Yes	783 (36.7)	5022 (59.2)
No	1353 (63.3)	3455 (40.8)
Surgery		
Yes	1994 (93.4)	8097 (95.5)
No	142 (6.6)	380 (4.5)

^a^including not receiving chemotherapy and unknown status

^b^including divorced, separated, single (never married), unmarried and widowed

^c^including married and domestic partner

^d^including White, American Indian/Alaskan native, Asian/Pacific Islander, and others-unspecified

**Fig. 5:**
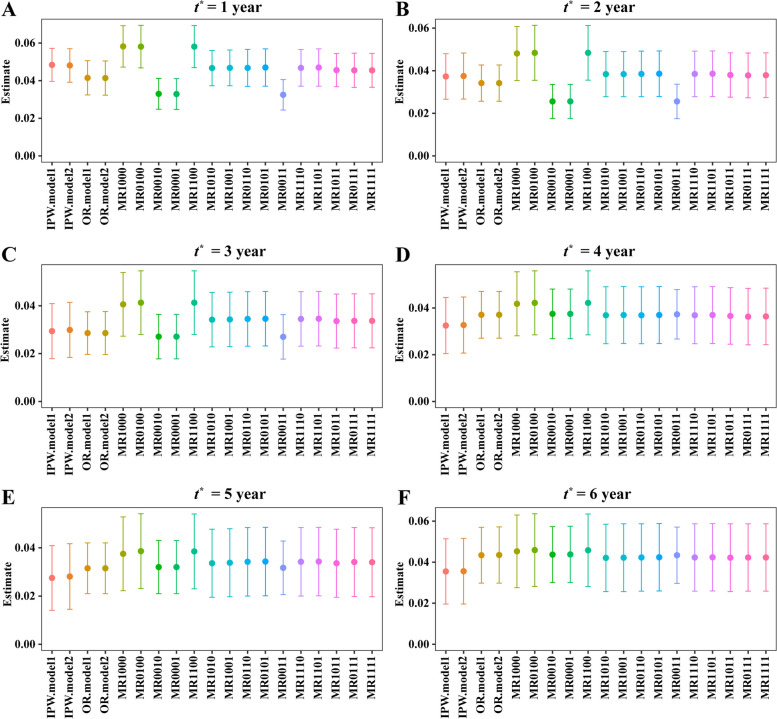
The triple-negative breast cancer data analysis: estimated $\Delta \left({t}^{*}\right)$ for ${t}^{*}=1, 2, 3, 4, 5$ and 6 years using different methods

To identify the candidate models for MR method, we explored the association of chemotherapy with all covariates through the multivariate logistic regression, and the association of survival with all covariates through the multivariate Cox model. Two sets of covariates for each model were identified using the significant level at 0.05 and 0.1. Two sets of covariates in $[\{{\pi }^{1}\left({\varvec{X}};{{\varvec{\beta}}}^{1}\right),{\pi }^{2}\left({\varvec{X}};{{\varvec{\beta}}}^{2}\right)]$ are: (i) marital status, grade, stage, age, radiotherapy, and surgery; (ii) marital status, race, grade, stage, age, radiotherapy, and surgery. Two sets of covariates in $\left[{m}^{1}\left({\varvec{X}},Z;{{\varvec{\gamma}}}^{1}\right), {m}^{2}\left({\varvec{X}},Z;{{\varvec{\gamma}}}^{2}\right)\right]$ are: (i) marital status, race, grade, stage, distant metastasis, age, radiotherapy, surgery, and chemotherapy; (ii) marital status, race, laterality, grade, stage, distant metastasis, age, radiotherapy, surgery, and chemotherapy. We applied the IPW, direct confounding adjustment, and MR methods to estimate the $\Delta \left(t\right)$ at the time point ${t}^{*}=\mathrm{year\;}1, 2, 3, 4, 5$ and 6. The results were shown in Fig. 5 and Table S5.

From Fig. 5 and Table S5, all estimators indicated that chemotherapy could improve the prognosis of TNBC. There were some differences among the values of different estimators. For example, the estimates of MR1000, MR0100 and MR1100 are larger than the other MR estimates at ${t}^{*}=\mathrm{year }1, 2, 3, 4, 5, 6$; and the estimates of MR0010, MR0001 and MR0000 seem to be smaller than the other MR estimates at ${t}^{*}=\mathrm{year }1, 2, 3$ year. Yet, the proposed method could include multiple PS models and OR models, which were more robust to the model specification to a certain extent and might yield more accurate estimates. And the estimated chemotherapy effects using MR1111 are 0.0455(0.0278–0.0633), 0.0379(0.0173–0.0586), 0.0337(0.0116–0.0559), 0.0364(0.0127–0.0601), 0.0340(0.0061–0.0620) and 0.0423(0.0101–0.0745) at t^*^= year 1, 2, 3, 4, 5, 6, respectively. The results suggests that the chemotherapy group had a survival rate of 4.55%, 3.79%, 3.37%, 3.64%, 3.40% and 4.23% higher than the controls at t^*^= year 1, 2, 3, 4, 5, 6, respectively; and the effects were all statistically significant at the 95% level.

## Discussion

In this study, we considered estimating the difference in survival functions between control and treatment groups in observational studies. The proposed MR method allowed multiple PS and OR models, and the bias of the proposed method is small when any model for propensity score or outcome regression is correctly specified regardless of in proportional or non-proportional hazard settings. Although we mainly focused on estimating the difference in survival functions in methodology, our proposed method can be easily extended to estimate other time-to-event parameters. For example, the restricted average survival causal effect ${\int }_{0}^{{t}^{*}}{S}^{1}\left(t\right)-{\int }_{0}^{{t}^{*}}{S}^{0}\left(t\right)$ compares the survival time at ${t}^{*}$, and the survival quantile effect ${\left({S}^{1}\right)}^{-1}\left(1-q\right)-{\left({S}^{0}\right)}^{-1}\left(1-q\right)$ compares the $q$-quantile of the survival time distribution between two groups [2].

Wang [4] combined the advantages of augmented inverse propensity weighted and PO method to proposed a simple, doubly robust method for estimating the causal effect of survival outcome [4]. Although the DR method also can avoid proportional hazards assumption, the DR method may lead to biased estimates when OR model and PS model are both misspecified [4]. By contrast, our proposed method could allow multiple PS models and multiple OR models, which provides extra protection from model misspecification. Our simulation studies showed that the IPW and direct confounding adjustment methods could lead to large bias and low coverage rate when misspecifying the propensity score and outcome regression models. By contrast, our proposed method showed satisfactory performance with finite sample sizes. For the proposed method, the bias was ignorable and the coverage rate was close to 95% when correctly specifying any PS and OR models.

There are some limitations in the study. First, we assume that there is no unmeasured confounding; However, there are often confounding variables that cannot be measured in observational studies, which leads to a biased estimation [23, 24]. Second, our proposed method could not deal with competing risks, which are also common in survival data [25, 26]. Third, we identify the candidate models based on the significance level of 0.05 and 0.1 in the empirical study, while the model specification may need clinical knowledge and existing literature, or statistical methods [27, 28]. Fourth, although the proposed method could multiple PS models and multiple OR models, which provides multiple protection against model misspecification, there may a biased estimated when the specified models do not include a correct model.

## Conclusions

Under the positivity, no unmeasured confounding (conditional exchangeability), consistency assumptions, the proposed MR method, pseudo-observation-based IPW and pseudo-observation-based direct confounding adjustment methods could be used to estimate the difference in survival functions. However, Pseudo-observation-based IPW or pseudo-observation-based direct confounding adjustment may lead to a biased estimate when the PS model or OR models is misspecified. By contrast, the proposed MR method based on the pseudo-observation approach has several advantages. First, the proposed method could allow a set of PS models and a set of OR models, and the proposed method has a small bias when any PS and OR models were correctly specified. Second. The proposed method considered the advantage of pseudo-observation approach, which avoids proportional hazards assumption. In actual research, the proposed estimator provided a new choice when researchers have a difficult time choosing only one model for their studies.

### Supplementary Information

**Additional file 1:** **Table S1.** Simulation results with different sample sizes =200 or 500 and different censoring rates =25% or 50% in the scenario where proportional hazard assumption holds based on 1000 replication. **Table S2.** Simulation results with different sample sizes =200 or 500 and different censoring rates =25% or 50% in the scenario where proportional hazard assumption dose not hold based on 1000 replication. **Table S3.** Simulation results with different sample sizes =200 or 500 and different censoring rates =25% or 50% in the scenario where proportional hazard assumption holds based on 1000 replication when PS models misspecified. **Table S4.** Simulation results with different sample sizes =200 or 500 and different censoring rates =25% or 50% in the scenario where proportional hazard assumption dose not hold based on 1000 replication when PS models misspecified. **Table S5.** The triple-negative breast cancer data analysis: estimated Δ (t^*^) for t^*^= year 1, 2, 3, 4, 5 and 6 years using different methods. **Figure S1.** Weibull survival functions defining simulation scenario. Figure A shows proportional hazards assumption holds; and Figure B shows that proportional hazards assumption dose not hold.

## Data Availability

Publicly available datasets were analyzed in this study. These data can be downloaded from https://seer.cancer.gov/. The simulation code can be downloaded from the github website    https://github.com/Ce-Wang/PO-MR.git).

## Abbreviations

ACE: Average causal effect
AJCC: American Joint Committee on Cancer
GEE: Generalized estimating equation
GLM: Generalized linear model
IPW: Inverse probability weighting
KM: Kaplan–Meier
MR: Multiply robust
OR: Outcome regression
PS: Propensity score
RMSE: Root mean square error
TNBC: Triple-negative breast cancer
