# Supplementary material for: Multiply robust estimator for the difference in survival functions using pseudo-observations
Source: BMC Med Res Methodol. 2023 Oct 23;23:247. doi: 10.1186/s12874-023-02065-6 (PMC10591363; doi:10.1186/s12874-023-02065-6)
Supplement: Supplementary file 1 — Additional file 1: Table S1. Simulation results with different sample sizes =200 or 500 and different censoring rates =25% or 50% in the scenario where proportional hazard assumption holds based on 1000 replication. Table S2. Simulation results with different sample sizes =200 or 500 and different censoring rates =25% or 50% in the scenario where proportional hazard assumption dose not hold based on 1000 replication. Table S3. Simulation results with different sample sizes =200 or 500 and different censoring rates =25% or 50% in the scenario where proportional hazard assumption holds based on 1000 replication when PS models misspecified. Table S4. Simulation results with different sample sizes =200 or 500 and different censoring rates =25% or 50% in the scenario where proportional hazard assumption dose not hold based on 1000 replication when PS models misspecified. Table S5. The triple-negative breast cancer data analysis: estimated Δ (t*) for t*= year 1, 2, 3, 4, 5 and 6 years using different methods. Figure S1. Weibull survival functions defining simulation scenario. Figure A shows proportional hazards assumption holds; and Figure B shows that proportional hazards assumption dose not hold. [file 12874_2023_2065_MOESM1_ESM.docx]

**Supplementary materials for “Multiply robust estimator for the difference in survival functions using pseudo-observations”**

Ce Wang^1*^, Kecheng Wei^1*^, Chen Huang^1^, Yongfu Yu^1,2^, Guoyou Qin^1,2^

^1^ Department of Biostatistics, Key Laboratory for Health Technology Assessment, National Commission of Health, Key Laboratory of Public Health Safety of Ministry of Education, School of Public Health, Fudan University, Shanghai, China

^2^ Shanghai Institute of Infectious Disease and Biosecurity, Shanghai, China

* These authors contributed equally.

**Correspondence to:** Guoyou Qin [gyqin@fudan.edu.cn](mailto:gyqin@fudan.edu.cn),Yongfu Yu [yu@fudan.edu.cn](mailto:yu@fudan.edu.cn)

**Table S1.** Simulation results with different sample sizes =200 or 500 and different censoring rates =25% or 50% in the scenario where proportional hazard assumption holds based on 1000 replication

| Sample size=200 | | | | | | | | | | | | | | | |
| --- | --- | --- | --- | --- | --- | --- | --- | --- | --- | --- | --- | --- | --- | --- | --- |
| Estimator | Censoring rate=25% | | | | | | |  | Censoring rate=50% | | | | | | |
|  | t=10 | | |  | t=20 | | |  | t=10 | | |  | t=20 | | |
|  | Bias(%) | RMSE | CR(%) |  | Bias(%) | RMSE | CR(%) |  | Bias(%) | RMSE | CR(%) |  | Bias(%) | RMSE | CR(%) |
| IPW.model1 | -0.6401 | 0.0617 | 95.0 |  | 0.0616 | 0.0744 | 95.1 |  | 0.1478 | 0.0723 | 94.2 |  | 1.1714 | 0.0992 | 94.2 |
| IPW.model2 | -38.9278 | 0.0821 | 83.0 |  | -34.7454 | 0.1227 | 76.3 |  | -38.0633 | 0.0880 | 85.3 |  | -33.6972 | 0.1371 | 82.9 |
| OR.model1 | 1.4270 | 0.0682 | 97.0 |  | -2.0628 | 0.0754 | 96.6 |  | 2.2583 | 0.0722 | 98.2 |  | -1.3748 | 0.1022 | 98.0 |
| OR.model2 | -38.1115 | 0.0834 | 90.4 |  | -33.0053 | 0.1222 | 81.8 |  | -36.5193 | 0.0895 | 95.0 |  | -31.2254 | 0.1402 | 90.5 |
| MR1000 | -1.2939 | 0.0592 | 94.1 |  | -0.0529 | 0.0726 | 94.7 |  | -0.5448 | 0.0701 | 93.4 |  | 1.0106 | 0.0979 | 94.2 |
| MR0100 | -38.9506 | 0.0820 | 81.9 |  | -34.7853 | 0.1227 | 75.8 |  | -38.0800 | 0.0879 | 84.4 |  | -33.7236 | 0.1372 | 82.2 |
| MR0010 | -1.2376 | 0.0574 | 94.0 |  | -0.3435 | 0.0713 | 94.8 |  | -0.7476 | 0.0678 | 94.1 |  | 0.8377 | 0.0955 | 94.2 |
| MR0001 | -38.5718 | 0.0816 | 82.2 |  | -34.3087 | 0.1217 | 76.5 |  | -37.7778 | 0.0875 | 84.5 |  | -33.251 | 0.1362 | 82.9 |
| MR1100 | -1.8151 | 0.0602 | 95.1 |  | -0.1202 | 0.0738 | 95.0 |  | -0.7955 | 0.0713 | 93.2 |  | 0.7880 | 0.0995 | 94.3 |
| MR1010 | -1.4414 | 0.05910 | 94.1 |  | -0.1119 | 0.0724 | 94.5 |  | -0.6404 | 0.0701 | 93.4 |  | 0.9562 | 0.0977 | 93.8 |
| MR1001 | -1.9778 | 0.0596 | 94.5 |  | -0.1034 | 0.0732 | 95.0 |  | -1.1939 | 0.0705 | 93.1 |  | 0.7094 | 0.0986 | 94.2 |
| MR0110 | -1.5400 | 0.0580 | 94.8 |  | -0.2964 | 0.0720 | 95.1 |  | -0.8702 | 0.0686 | 94.3 |  | 0.7800 | 0.0968 | 94.5 |
| MR0101 | -38.8928 | 0.0819 | 81.9 |  | -34.7767 | 0.1228 | 76.1 |  | -38.0348 | 0.0878 | 85.2 |  | -33.6647 | 0.1373 | 82.4 |
| MR0011 | -2.2219 | 0.0580 | 94.6 |  | -0.3547 | 0.0715 | 94.5 |  | -1.6717 | 0.0681 | 94.2 |  | 0.5990 | 0.0961 | 93.9 |
| MR1110 | -1.7920 | 0.0599 | 95.0 |  | -0.1527 | 0.0734 | 94.9 |  | -0.7055 | 0.0713 | 93.3 |  | 0.7559 | 0.0990 | 94.3 |
| MR1101 | -2.4734 | 0.0602 | 95.3 |  | -0.4517 | 0.0734 | 95.3 |  | -1.5740 | 0.0714 | 93.0 |  | 0.4718 | 0.0991 | 94.4 |
| MR1011 | -1.8812 | 0.0594 | 94.4 |  | -0.2362 | 0.0731 | 94.7 |  | -0.8878 | 0.0705 | 93.2 |  | 0.6722 | 0.0982 | 93.9 |
| MR0111 | -2.2311 | 0.0583 | 95.0 |  | -0.4315 | 0.0716 | 94.9 |  | -1.5703 | 0.0687 | 94.3 |  | 0.5335 | 0.0967 | 94.3 |
| MR1111 | -2.1277 | 0.0600 | 95.0 |  | -0.3608 | 0.0732 | 95.1 |  | -1.0877 | 0.0715 | 92.9 |  | 0.5573 | 0.0988 | 94.5 |
| Sample size=500 | | | | | | | | | | | | | | | |
| Estimator | Censoring rate=25% | | | | | | |  | Censoring rate=50% | | | | | | |
|  | t=10 | | |  | t=20 | | |  | t=10 | | |  | t=20 | | |
|  | Bias(%) | RMSE | CR(%) |  | Bias(%) | RMSE | CR(%) |  | Bias(%) | RMSE | CR(%) |  | Bias(%) | RMSE | CR(%) |
| IPW.model1 | 1.1491 | 0.0375 | 95.4 |  | 0.7344 | 0.0454 | 94.7 |  | 0.8987 | 0.0431 | 94.8 |  | 1.0655 | 0.0625 | 94.7 |
| IPW.model2 | -36.4737 | 0.0648 | 68.5 |  | -34.4774 | 0.1053 | 50.3 |  | -36.6915 | 0.0679 | 73.7 |  | -34.3651 | 0.1122 | 67.3 |
| OR.model1 | 4.5384 | 0.0382 | 96.0 |  | -2.1480 | 0.0481 | 94.8 |  | 4.5295 | 0.0443 | 95.6 |  | -1.9497 | 0.0630 | 94.3 |
| OR.model2 | -37.3491 | 0.0665 | 69.2 |  | -33.9391 | 0.1058 | 56.8 |  | -37.4434 | 0.0695 | 75.1 |  | -33.5102 | 0.1132 | 72.0 |
| MR1000 | 0.9175 | 0.0365 | 96.0 |  | 0.7722 | 0.0450 | 94.5 |  | 0.6784 | 0.0421 | 95.3 |  | 1.0963 | 0.0619 | 94.5 |
| MR0100 | -36.4943 | 0.0649 | 67.7 |  | -34.4891 | 0.1054 | 50.1 |  | -36.7124 | 0.0680 | 73.3 |  | -34.381 | 0.1122 | 67.1 |
| MR0010 | 1.6302 | 0.0360 | 95.0 |  | 0.2777 | 0.0441 | 94.5 |  | 1.5160 | 0.0417 | 94.2 |  | 0.5947 | 0.0603 | 94.8 |
| MR0001 | -36.3973 | 0.0647 | 68.3 |  | -34.3142 | 0.1049 | 51.0 |  | -36.6033 | 0.0678 | 73.6 |  | -34.2384 | 0.1119 | 67.0 |
| MR1100 | 1.1699 | 0.0368 | 96.1 |  | 0.6948 | 0.0449 | 94.6 |  | 0.9420 | 0.0425 | 94.9 |  | 0.8900 | 0.062 | 95.0 |
| MR1010 | 1.0197 | 0.0364 | 95.3 |  | 0.7789 | 0.0449 | 94.2 |  | 0.7827 | 0.0420 | 94.7 |  | 1.0905 | 0.0616 | 94.6 |
| MR1001 | 0.8473 | 0.0367 | 96.0 |  | 0.7177 | 0.0450 | 94.8 |  | 0.6022 | 0.0424 | 94.7 |  | 0.9421 | 0.0621 | 94.4 |
| MR0110 | 1.6109 | 0.0363 | 95.1 |  | 0.3588 | 0.0441 | 94.4 |  | 1.4640 | 0.0419 | 95.1 |  | 0.614 | 0.0605 | 94.5 |
| MR0101 | -36.4902 | 0.0649 | 67.6 |  | -34.4799 | 0.1053 | 50.2 |  | -36.6996 | 0.0679 | 73.5 |  | -34.3689 | 0.1122 | 67.0 |
| MR0011 | 0.9997 | 0.0363 | 94.8 |  | 0.3195 | 0.0441 | 94.8 |  | 0.8009 | 0.0418 | 95.2 |  | 0.5648 | 0.0604 | 94.6 |
| MR1110 | 1.2140 | 0.0367 | 95.4 |  | 0.7669 | 0.0447 | 94.5 |  | 0.9684 | 0.0423 | 94.5 |  | 0.9647 | 0.0617 | 94.5 |
| MR1101 | 0.9687 | 0.0368 | 95.9 |  | 0.5862 | 0.0447 | 94.5 |  | 0.7219 | 0.0425 | 94.8 |  | 0.7513 | 0.0619 | 94.6 |
| MR1011 | 1.0010 | 0.0367 | 95.5 |  | 0.7426 | 0.0449 | 94.6 |  | 0.7301 | 0.0423 | 94.9 |  | 0.9789 | 0.0618 | 94.4 |
| MR0111 | 1.1521 | 0.0363 | 95.0 |  | 0.3550 | 0.0440 | 94.6 |  | 0.9787 | 0.0419 | 95.4 |  | 0.5903 | 0.0605 | 94.6 |
| MR1111 | 1.1270 | 0.0367 | 95.2 |  | 0.6965 | 0.0447 | 94.5 |  | 0.8498 | 0.0424 | 94.5 |  | 0.8501 | 0.0616 | 94.6 |

The true values of $\Delta\left( t=10 \right)$ or $\Delta\left( t=20 \right)$ are 0.1480 and 0.2725, respectively.

Bias (%): mean relative bias; RMSE: root mean square error; CR: coverage rate; IPW: inverse probability weighting; OR: outcome regression; MR, multiply robust; MR estimators are denoted as “MR-0000”, where each digit of the four numbers, from left to right, indicates if $\pi^{1}\left( \boldsymbol{X};\boldsymbol{\beta}^{1} \right)$, $\pi^{2}\left( \boldsymbol{X};\boldsymbol{\beta}^{2} \right)$, $m^{1}\left( \boldsymbol{X,}Z;\boldsymbol{\gamma}^{1} \right)$ or $m^{2}\left( \boldsymbol{X,}Z;\boldsymbol{\gamma}^{2} \right)$ is included in the estimator (“1” means yes and “0” means no).

**Table S2.** Simulation results with different sample sizes =200 or 500 and different censoring rates =25% or 50% in the scenario where proportional hazard assumption dose not hold based on 1000 replication

| Sample size=200 | | | | | | | | | | | | | | | |  |
| --- | --- | --- | --- | --- | --- | --- | --- | --- | --- | --- | --- | --- | --- | --- | --- | --- |
| Estimator | Censoring rate=25% | | | | | | |  | Censoring rate=50% | | | | | | |  |
|  | t=10 | | |  | t=20 | | |  | t=10 | | |  | t=20 | | |  |
|  | Bias(%) | RMSE | CR(%) |  | Bias(%) | RMSE | CR(%) |  | Bias(%) | RMSE | CR(%) |  | Bias(%) | RMSE | CR(%) | |
| IPW.model1 | -0.2814 | 0.0629 | 95.7 |  | -0.1767 | 0.0681 | 95.1 |  | 0.3948 | 0.0739 | 95.0 |  | 0.6300 | 0.0899 | 95.7 | |
| IPW.model2 | -26.2514 | 0.0906 | 82.5 |  | -20.7552 | 0.1056 | 78.7 |  | -25.4846 | 0.0961 | 85.1 |  | -20.0315 | 0.1181 | 85.5 | |
| OR.model1 | 2.7498 | 0.0638 | 97.1 |  | -2.4527 | 0.0723 | 97.5 |  | 3.0914 | 0.0787 | 98.2 |  | -2.2283 | 0.0998 | 99.3 | |
| OR.model2 | -24.7687 | 0.0895 | 85.6 |  | -18.5822 | 0.1028 | 88.4 |  | -23.8756 | 0.1000 | 90.5 |  | -18.9728 | 0.1533 | 94.8 | |
| MR1000 | -0.6073 | 0.0609 | 95.3 |  | -0.2965 | 0.0657 | 94.5 |  | 0.0256 | 0.0720 | 94.0 |  | 0.4984 | 0.0878 | 94.7 | |
| MR0100 | -26.2591 | 0.0905 | 81.4 |  | -20.7736 | 0.1056 | 77.9 |  | -25.4894 | 0.0959 | 84.8 |  | -20.0307 | 0.1182 | 84.7 | |
| MR0010 | -0.9431 | 0.0587 | 95.4 |  | -0.1840 | 0.0645 | 94.8 |  | -0.3070 | 0.0688 | 94.7 |  | 0.4578 | 0.0861 | 95.5 | |
| MR0001 | -25.9829 | 0.0900 | 81.9 |  | -20.5239 | 0.1048 | 78.2 |  | -25.2764 | 0.0953 | 84.7 |  | -19.7659 | 0.1171 | 85.9 | |
| MR1100 | -0.8241 | 0.0619 | 96.7 |  | -0.2920 | 0.0671 | 94.3 |  | -0.1863 | 0.0731 | 94.3 |  | 0.3610 | 0.0893 | 95.7 | |
| MR1010 | -0.6626 | 0.0606 | 95.2 |  | -0.2659 | 0.0656 | 94.7 |  | 0.0215 | 0.0718 | 94.4 |  | 0.5486 | 0.0876 | 95.2 | |
| MR1001 | -0.7930 | 0.0614 | 95.6 |  | -0.3056 | 0.0661 | 95.0 |  | -0.3115 | 0.0726 | 93.8 |  | 0.2853 | 0.0881 | 95.4 | |
| MR0110 | -1.0382 | 0.0596 | 95.1 |  | -0.2050 | 0.0652 | 94.8 |  | -0.4902 | 0.0699 | 94.8 |  | 0.3552 | 0.0872 | 95.6 | |
| MR0101 | -26.2268 | 0.0904 | 81.6 |  | -20.7393 | 0.1056 | 78.0 |  | -25.4515 | 0.0958 | 84.9 |  | -19.989 | 0.1183 | 85.0 | |
| MR0011 | -1.1404 | 0.0595 | 95.3 |  | -0.2707 | 0.0649 | 94.6 |  | -0.6600 | 0.0698 | 94.4 |  | 0.2572 | 0.0862 | 96.0 | |
| MR1110 | -0.8492 | 0.0617 | 95.7 |  | -0.3102 | 0.0666 | 94.3 |  | -0.1665 | 0.0730 | 94.3 |  | 0.3757 | 0.0888 | 95.6 | |
| MR1101 | -1.1663 | 0.0621 | 96.4 |  | -0.5262 | 0.0669 | 94.5 |  | -0.6161 | 0.0733 | 94.3 |  | 0.1210 | 0.0894 | 96.2 | |
| MR1011 | -0.8118 | 0.0611 | 95.1 |  | -0.3423 | 0.0661 | 94.5 |  | -0.2411 | 0.0725 | 93.9 |  | 0.2885 | 0.0878 | 95.4 | |
| MR0111 | -1.2191 | 0.0600 | 95.0 |  | -0.3460 | 0.065 | 94.8 |  | -0.7243 | 0.0704 | 94.6 |  | 0.1656 | 0.0867 | 95.8 | |
| MR1111 | -1.0171 | 0.0620 | 95.7 |  | -0.4800 | 0.0664 | 94.3 |  | -0.4247 | 0.0732 | 94.1 |  | 0.2082 | 0.0886 | 96.0 | |
| Sample size=500 | | | | | | | | | | | | | | | |  |
| Estimator | Censoring rate=25% | | | | | | |  | Censoring rate=50% | | | | | | |  |
|  | t=10 | | |  | t=20 | | |  | t=10 | | |  | t=20 | | |  |
|  | Bias(%) | RMSE | CR(%) |  | Bias(%) | RMSE | CR(%) |  | Bias(%) | RMSE | CR(%) |  | Bias(%) | RMSE | CR(%) | |
| IPW.model1 | 0.9376 | 0.0391 | 95.5 |  | 0.2674 | 0.0411 | 95.1 |  | 0.7618 | 0.0458 | 94.4 |  | 0.4845 | 0.0567 | 94.4 | |
| IPW.model2 | -24.7409 | 0.0735 | 64.6 |  | -20.3538 | 0.0883 | 55.6 |  | -25.0191 | 0.0773 | 70.3 |  | -20.2591 | 0.0947 | 71.7 | |
| OR.model1 | 3.8864 | 0.0406 | 95.1 |  | -2.5049 | 0.0460 | 94.4 |  | 3.8334 | 0.0476 | 94.8 |  | -2.0970 | 0.0602 | 95.2 | |
| OR.model2 | -24.5363 | 0.0737 | 67.3 |  | -17.9955 | 0.0827 | 68.7 |  | -24.6226 | 0.0772 | 73.1 |  | -17.8659 | 0.0904 | 82.2 | |
| MR1000 | 0.8581 | 0.0382 | 95.9 |  | 0.2741 | 0.0405 | 95.2 |  | 0.6953 | 0.0449 | 94.4 |  | 0.4847 | 0.0559 | 93.8 | |
| MR0100 | -24.7519 | 0.0735 | 64.6 |  | -20.3609 | 0.0883 | 55.3 |  | -25.0308 | 0.0773 | 70.5 |  | -20.2668 | 0.0948 | 71.8 | |
| MR0010 | 0.8791 | 0.0375 | 94.8 |  | 0.4286 | 0.0400 | 95.3 |  | 0.6967 | 0.0440 | 93.6 |  | 0.6053 | 0.0544 | 93.7 | |
| MR0001 | -24.6733 | 0.0733 | 64.9 |  | -20.2862 | 0.0880 | 56.4 |  | -24.9447 | 0.0771 | 70.7 |  | -20.1677 | 0.0943 | 71.2 | |
| MR1100 | 1.0304 | 0.0386 | 95.5 |  | 0.2317 | 0.0407 | 95.5 |  | 0.8697 | 0.0453 | 94.8 |  | 0.3449 | 0.0562 | 94.0 | |
| MR1010 | 0.8853 | 0.0381 | 95.3 |  | 0.2877 | 0.0405 | 95.0 |  | 0.7324 | 0.0448 | 94.4 |  | 0.4808 | 0.0557 | 94.0 | |
| MR1001 | 0.8558 | 0.0383 | 95.5 |  | 0.2120 | 0.0406 | 95.1 |  | 0.6765 | 0.0451 | 94.5 |  | 0.3598 | 0.0563 | 94.2 | |
| MR0110 | 0.9981 | 0.0378 | 94.6 |  | 0.3992 | 0.0401 | 95.4 |  | 0.7992 | 0.0442 | 94.3 |  | 0.5221 | 0.0548 | 94.1 | |
| MR0101 | -24.7411 | 0.0735 | 64.6 |  | -20.3554 | 0.0883 | 55.6 |  | -25.0174 | 0.0773 | 70.6 |  | -20.252 | 0.0947 | 71.7 | |
| MR0011 | 0.8138 | 0.0378 | 94.4 |  | 0.3335 | 0.0398 | 95.5 |  | 0.5850 | 0.0443 | 94.3 |  | 0.4763 | 0.0545 | 93.9 | |
| MR1110 | 1.0351 | 0.0384 | 95.1 |  | 0.2762 | 0.0406 | 95.3 |  | 0.8797 | 0.0451 | 94.5 |  | 0.3775 | 0.0561 | 94.1 | |
| MR1101 | 0.9383 | 0.0386 | 95.6 |  | 0.1680 | 0.0407 | 95.3 |  | 0.7608 | 0.0453 | 95.0 |  | 0.2464 | 0.0563 | 94.1 | |
| MR1011 | 0.8917 | 0.0383 | 95.1 |  | 0.2359 | 0.0406 | 95.0 |  | 0.7279 | 0.0450 | 94.5 |  | 0.3611 | 0.0561 | 94.0 | |
| MR0111 | 0.9284 | 0.0379 | 94.5 |  | 0.2969 | 0.0400 | 95.6 |  | 0.7086 | 0.0445 | 94.0 |  | 0.4110 | 0.0546 | 94.1 | |
| MR1111 | 0.9974 | 0.0385 | 95.1 |  | 0.2066 | 0.0406 | 95.2 |  | 0.8136 | 0.0451 | 94.6 |  | 0.2906 | 0.0561 | 94.4 | |

The true values of $\Delta\left( t=10 \right)$ or $\Delta\left( t=20 \right)$ are 0.2524 and 0.3816, respectively

Bias (%): mean relative bias; RMSE: root mean square error; CR: coverage rate; IPW: inverse probability weighting; OR: outcome regression; MR, multiply robust. MR estimators are denoted as “MR-0000”, where each digit of the four numbers, from left to right, indicates if $\pi^{1}\left( \boldsymbol{X};\boldsymbol{\beta}^{1} \right)$, $\pi^{2}\left( \boldsymbol{X};\boldsymbol{\beta}^{2} \right)$, $m^{1}\left( \boldsymbol{X,}Z;\boldsymbol{\gamma}^{1} \right)$ or $m^{2}\left( \boldsymbol{X,}Z;\boldsymbol{\gamma}^{2} \right)$ is included in the estimator (“1” means yes and “0” means no).

**Table S3.** Simulation results with different sample sizes =200 or 500 and different censoring rates =25% or 50% in the scenario where proportional hazard assumption holds based on 1000 replication when PS models misspecified

| **Sample size=200** | | | | | | | | | | | | | | | |  |
| --- | --- | --- | --- | --- | --- | --- | --- | --- | --- | --- | --- | --- | --- | --- | --- | --- |
| Estimator | Censoring rate=25% | | | | | | |  | Censoring rate=50% | | | | | | |  |
|  | t=10 | | |  | t=20 | | |  | t=10 | | |  | t=20 | | |  |
|  | Bias(%) | RMSE | CR(%) |  | Bias(%) | RMSE | CR(%) |  | Bias(%) | RMSE | CR(%) |  | Bias(%) | RMSE | CR(%) | |
| IPW.model1 | -38.785 | 0.0818 | 82.5 |  | -34.6201 | 0.1225 | 76.2 |  | -37.9504 | 0.0877 | 84.6 |  | -33.4922 | 0.1366 | 82.6 | |
| IPW.model2 | -38.9278 | 0.0821 | 83.0 |  | -34.7454 | 0.1227 | 76.3 |  | -38.0633 | 0.088 | 85.3 |  | -33.6972 | 0.1371 | 82.9 | |
| OR.model1 | 1.4270 | 0.0682 | 97.0 |  | -2.0628 | 0.0754 | 96.6 |  | 2.2583 | 0.0722 | 98.2 |  | -1.3748 | 0.1022 | 98.0 | |
| OR.model2 | -38.1115 | 0.0834 | 90.4 |  | -33.0053 | 0.1222 | 81.8 |  | -36.5193 | 0.0895 | 95.0 |  | -31.2254 | 0.1402 | 90.5 | |
| MR1000 | -38.8597 | 0.0819 | 81.4 |  | -34.6886 | 0.1226 | 75.8 |  | -38.0297 | 0.0877 | 84.5 |  | -33.5328 | 0.1367 | 82.3 | |
| MR0100 | -38.9506 | 0.0820 | 81.9 |  | -34.7853 | 0.1227 | 75.8 |  | -38.08 | 0.0879 | 84.4 |  | -33.7236 | 0.1372 | 82.2 | |
| MR0010 | -1.2376 | 0.0574 | 94.0 |  | -0.3435 | 0.0713 | 94.8 |  | -0.7476 | 0.0678 | 94.1 |  | 0.8377 | 0.0955 | 94.2 | |
| MR0001 | -38.5718 | 0.0816 | 82.2 |  | -34.3087 | 0.1217 | 76.5 |  | -37.7778 | 0.0875 | 84.5 |  | -33.251 | 0.1362 | 82.9 | |
| MR1100 | -38.9464 | 0.0820 | 81.9 |  | -34.8137 | 0.1229 | 75.6 |  | -38.0756 | 0.0879 | 84.5 |  | -33.7435 | 0.1373 | 82.4 | |
| MR1010 | -1.5405 | 0.0579 | 94.6 |  | -0.1897 | 0.0717 | 94.8 |  | -0.9476 | 0.0685 | 94.2 |  | 0.9634 | 0.0965 | 94.1 | |
| MR1001 | -38.8755 | 0.0819 | 82.0 |  | -34.6969 | 0.1226 | 75.8 |  | -38.0427 | 0.0878 | 84.8 |  | -33.531 | 0.137 | 82.3 | |
| MR0110 | -1.5400 | 0.058 | 94.8 |  | -0.2964 | 0.0720 | 95.1 |  | -0.8702 | 0.0686 | 94.3 |  | 0.78 | 0.0968 | 94.5 | |
| MR0101 | -38.8928 | 0.0819 | 81.9 |  | -34.7767 | 0.1228 | 76.1 |  | -38.0348 | 0.0878 | 85.2 |  | -33.6647 | 0.1373 | 82.4 | |
| MR0011 | -2.2219 | 0.0580 | 94.6 |  | -0.3547 | 0.0715 | 94.5 |  | -1.6717 | 0.0681 | 94.2 |  | 0.599 | 0.0961 | 93.9 | |
| MR1110 | -1.6800 | 0.0579 | 95.0 |  | -0.2523 | 0.0718 | 95.4 |  | -1.0843 | 0.0684 | 94.6 |  | 0.8068 | 0.0968 | 94.3 | |
| MR1101 | -38.973 | 0.0820 | 82.0 |  | -34.8272 | 0.1229 | 76.1 |  | -38.066 | 0.0879 | 85.1 |  | -33.7331 | 0.1374 | 82.5 | |
| MR1011 | -1.7693 | 0.0583 | 95.0 |  | -0.137 | 0.0713 | 95.1 |  | -1.2094 | 0.0686 | 94.5 |  | 0.8905 | 0.0967 | 93.9 | |
| MR0111 | -2.2311 | 0.0583 | 95.0 |  | -0.4315 | 0.0716 | 94.9 |  | -1.5703 | 0.0687 | 94.3 |  | 0.5335 | 0.0967 | 94.3 | |
| MR1111 | -1.9176 | 0.0583 | 95.4 |  | -0.2055 | 0.0714 | 95.2 |  | -1.3034 | 0.0686 | 94.4 |  | 0.7702 | 0.0969 | 94.0 | |
| **Sample size=500** | | | | | | | | | | | | | | | |  |
| Estimator | Censoring rate=25% | | | | | | |  | Censoring rate=50% | | | | | | |  |
|  | t=10 | | |  | t=20 | | |  | t=10 | | |  | t=20 | | |  |
|  | Bias(%) | RMSE | CR(%) |  | Bias(%) | RMSE | CR(%) |  | Bias(%) | RMSE | CR(%) |  | Bias(%) | RMSE | CR(%) | |
| IPW.model1 | -36.5136 | 0.0649 | 68.2 |  | -34.5019 | 0.1054 | 50.2 |  | -36.7193 | 0.0679 | 73.8 |  | -34.4069 | 0.1122 | 66.7 | |
| IPW.model2 | -36.4737 | 0.0648 | 68.5 |  | -34.4774 | 0.1053 | 50.3 |  | -36.6915 | 0.0679 | 73.7 |  | -34.3651 | 0.1122 | 67.3 | |
| OR.model1 | 4.5384 | 0.0382 | 96.0 |  | -2.1480 | 0.0481 | 94.8 |  | 4.5295 | 0.0443 | 95.6 |  | -1.9497 | 0.0630 | 94.3 | |
| OR.model2 | -37.3491 | 0.0665 | 69.2 |  | -33.9391 | 0.1058 | 56.8 |  | -37.4434 | 0.0695 | 75.1 |  | -33.5102 | 0.1132 | 72.0 | |
| MR1000 | -36.5427 | 0.0649 | 67.6 |  | -34.5228 | 0.1055 | 50.4 |  | -36.7479 | 0.0680 | 73.2 |  | -34.4325 | 0.1123 | 66.3 | |
| MR0100 | -36.4943 | 0.0649 | 67.7 |  | -34.4891 | 0.1054 | 50.1 |  | -36.7124 | 0.0680 | 73.3 |  | -34.381 | 0.1122 | 67.1 | |
| MR0010 | 1.6302 | 0.036 | 95.0 |  | 0.2777 | 0.0441 | 94.5 |  | 1.5160 | 0.0417 | 94.2 |  | 0.5947 | 0.0603 | 94.8 | |
| MR0001 | -36.3973 | 0.0647 | 68.3 |  | -34.3142 | 0.1049 | 51.0 |  | -36.6033 | 0.0678 | 73.6 |  | -34.2384 | 0.1119 | 67.0 | |
| MR1100 | -36.498 | 0.0649 | 67.8 |  | -34.499 | 0.1054 | 50.2 |  | -36.7168 | 0.0680 | 73.1 |  | -34.3935 | 0.1123 | 67.0 | |
| MR1010 | 1.4308 | 0.0362 | 95.2 |  | 0.3541 | 0.0441 | 94.6 |  | 1.2411 | 0.0418 | 95.0 |  | 0.5846 | 0.0604 | 94.6 | |
| MR1001 | -36.5324 | 0.0649 | 67.9 |  | -34.5147 | 0.1054 | 50.7 |  | -36.7439 | 0.0680 | 73.4 |  | -34.413 | 0.1123 | 67.2 | |
| MR0110 | 1.6109 | 0.0363 | 95.1 |  | 0.3588 | 0.0441 | 94.4 |  | 1.4640 | 0.0419 | 95.1 |  | 0.614.0 | 0.0605 | 94.5 | |
| MR0101 | -36.4902 | 0.0649 | 67.6 |  | -34.4799 | 0.1053 | 50.2 |  | -36.6996 | 0.0679 | 73.5 |  | -34.3689 | 0.1122 | 67.0 | |
| MR0011 | 0.9997 | 0.0363 | 94.8 |  | 0.3195 | 0.0441 | 94.8 |  | 0.8009 | 0.0418 | 95.2 |  | 0.5648 | 0.0604 | 94.6 | |
| MR1110 | 1.4964 | 0.0362 | 95.3 |  | 0.3818 | 0.0440 | 94.5 |  | 1.3094 | 0.0418 | 94.9 |  | 0.6238 | 0.0605 | 94.3 | |
| MR1101 | -36.4888 | 0.0649 | 68.0 |  | -34.4919 | 0.1054 | 50.3 |  | -36.7165 | 0.0680 | 73.3 |  | -34.3733 | 0.1123 | 67.2 | |
| MR1011 | 1.2093 | 0.0363 | 94.7 |  | 0.3885 | 0.0440 | 94.6 |  | 1.0263 | 0.0420 | 94.9 |  | 0.6434 | 0.0604 | 94.5 | |
| MR0111 | 1.1521 | 0.0363 | 95.0 |  | 0.3550 | 0.0440 | 94.6 |  | 0.9787 | 0.0419 | 95.4 |  | 0.5903 | 0.0605 | 94.6 | |
| MR1111 | 1.2747 | 0.0362 | 94.9 |  | 0.4139 | 0.0440 | 94.6 |  | 1.0913 | 0.0420 | 95.1 |  | 0.6809 | 0.0605 | 94.3 | |

The true values of $\Delta\left( t=10 \right)$ or $\Delta\left( t=20 \right)$ are 0.1480 and 0.2725, respectively.

Bias (%): mean relative bias; RMSE: root mean square error; CR: coverage rate; IPW: inverse probability weighting; OR: outcome regression; MR, multiply robust. MR estimators are denoted as “MR-0000”, where each digit of the four numbers, from left to right, indicates if $\pi^{1}\left( \boldsymbol{X};\boldsymbol{\beta}^{1} \right)$, $\pi^{2}\left( \boldsymbol{X};\boldsymbol{\beta}^{2} \right)$, $m^{1}\left( \boldsymbol{X,}Z;\boldsymbol{\gamma}^{1} \right)$ or $m^{2}\left( \boldsymbol{X,}Z;\boldsymbol{\gamma}^{2} \right)$ is included in the estimator (“1” means yes and “0” means no).

**Table S4**. Simulation results with different sample sizes =200 or 500 and different censoring rates =25% or 50% in the scenario where proportional hazard assumption dose not hold based on 1000 replication when PS models misspecified

| **Sample size=200** | | | | | | | | | | | | | | | |  |
| --- | --- | --- | --- | --- | --- | --- | --- | --- | --- | --- | --- | --- | --- | --- | --- | --- |
| Estimator | Censoring rate=25% | | | | | | |  | Censoring rate=50% | | | | | | |  |
|  | t=10 | | |  | t=20 | | |  | t=10 | | |  | t=20 | | |  |
|  | Bias(%) | RMSE | CR(%) |  | Bias(%) | RMSE | CR(%) |  | Bias(%) | RMSE | CR(%) |  | Bias(%) | RMSE | CR(%) | |
| IPW.model1 | -26.1476 | 0.0903 | 82.1 |  | -20.6736 | 0.1053 | 79.0 |  | -25.3623 | 0.0957 | 85.1 |  | -19.9411 | 0.1178 | 85.5 | |
| IPW.model2 | -26.2514 | 0.0906 | 82.5 |  | -20.7552 | 0.1056 | 78.7 |  | -25.4846 | 0.0961 | 85.1 |  | -20.0315 | 0.1181 | 85.5 | |
| OR.model1 | 2.7498 | 0.0638 | 97.1 |  | -2.4527 | 0.0723 | 97.5 |  | 3.0914 | 0.0787 | 98.2 |  | -2.2283 | 0.0998 | 99.3 | |
| OR.model2 | -24.7687 | 0.0895 | 85.6 |  | -18.5822 | 0.1028 | 88.4 |  | -23.8756 | 0.100 | 90.5 |  | -18.9728 | 0.1533 | 94.8 | |
| MR1000 | -26.1925 | 0.0903 | 81.2 |  | -20.7073 | 0.1053 | 78.5 |  | -25.4129 | 0.0957 | 84.7 |  | -19.9576 | 0.1178 | 85.2 | |
| MR0100 | -26.2591 | 0.0905 | 81.4 |  | -20.7736 | 0.1056 | 77.9 |  | -25.4894 | 0.0959 | 84.8 |  | -20.0307 | 0.1182 | 84.7 | |
| MR0010 | -0.9431 | 0.0587 | 95.4 |  | -0.1840 | 0.0645 | 94.8 |  | -0.3070 | 0.0688 | 94.7 |  | 0.4578 | 0.0861 | 95.5 | |
| MR0001 | -25.9829 | 0.0900 | 81.9 |  | -20.5239 | 0.1048 | 78.2 |  | -25.2764 | 0.0953 | 84.7 |  | -19.7659 | 0.1171 | 85.9 | |
| MR1100 | -26.2495 | 0.0906 | 81.4 |  | -20.7849 | 0.1056 | 77.8 |  | -25.4739 | 0.0959 | 84.9 |  | -20.0444 | 0.1182 | 84.9 | |
| MR1010 | -0.9342 | 0.0595 | 95.1 |  | -0.1670 | 0.0650 | 94.7 |  | -0.3814 | 0.0699 | 95.1 |  | 0.4591 | 0.0868 | 95.6 | |
| MR1001 | -26.1752 | 0.0903 | 81.8 |  | -20.6800 | 0.1053 | 78.8 |  | -25.4141 | 0.0956 | 84.8 |  | -19.9386 | 0.1179 | 85.6 | |
| MR0110 | -1.0382 | 0.0596 | 95.1 |  | -0.2050 | 0.0652 | 94.8 |  | -0.4902 | 0.0699 | 94.8 |  | 0.3552 | 0.0872 | 95.6 | |
| MR0101 | -26.2268 | 0.0904 | 81.6 |  | -20.7393 | 0.1056 | 78.0 |  | -25.4515 | 0.0958 | 84.9 |  | -19.989 | 0.1183 | 85.0 | |
| MR0011 | -1.1404 | 0.0595 | 95.3 |  | -0.2707 | 0.0649 | 94.6 |  | -0.6600 | 0.0698 | 94.4 |  | 0.2572 | 0.0862 | 96.0 | |
| MR1110 | -1.0436 | 0.0598 | 95.1 |  | -0.2025 | 0.0650 | 94.8 |  | -0.5311 | 0.0701 | 94.7 |  | 0.3677 | 0.0870 | 95.9 | |
| MR1101 | -26.2315 | 0.0904 | 81.7 |  | -20.7657 | 0.1056 | 78.4 |  | -25.4861 | 0.0959 | 84.9 |  | -20.0535 | 0.1186 | 85.1 | |
| MR1011 | -0.9663 | 0.0600 | 95.3 |  | -0.2125 | 0.0650 | 94.6 |  | -0.3821 | 0.0703 | 94.8 |  | 0.2695 | 0.0867 | 96.0 | |
| MR0111 | -1.2191 | 0.0600 | 95.0 |  | -0.3460 | 0.0650 | 94.8 |  | -0.7243 | 0.0704 | 94.6 |  | 0.1656 | 0.0867 | 95.8 | |
| MR1111 | -1.0740 | 0.0602 | 95.2 |  | -0.2643 | 0.0650 | 94.8 |  | -0.5335 | 0.0705 | 94.7 |  | 0.1926 | 0.0868 | 95.9 | |
| **Sample size=500** | | | | | | | | | | | | | | | |  |
| Estimator | Censoring rate=25% | | | | | | |  | Censoring rate=50% | | | | | | |  |
|  | t=10 | | |  | t=20 | | |  | t=10 | | |  | t=20 | | |  |
|  | Bias(%) | RMSE | CR(%) |  | Bias(%) | RMSE | CR(%) |  | Bias(%) | RMSE | CR(%) |  | Bias(%) | RMSE | CR(%) | |
| IPW.model1 | -24.7554 | 0.0735 | 64.7 |  | -20.3559 | 0.0883 | 56.1 |  | -25.0189 | 0.0772 | 70.7 |  | -20.2599 | 0.0947 | 71.6 | |
| IPW.model2 | -24.7409 | 0.0735 | 64.6 |  | -20.3538 | 0.0883 | 55.6 |  | -25.0191 | 0.0773 | 70.3 |  | -20.2591 | 0.0947 | 71.7 | |
| OR.model1 | 3.8864 | 0.0406 | 95.1 |  | -2.5049 | 0.046 | 94.4 |  | 3.8334 | 0.0476 | 94.8 |  | -2.0970 | 0.0602 | 95.2 | |
| OR.model2 | -24.5363 | 0.0737 | 67.3 |  | -17.9955 | 0.0827 | 68.7 |  | -24.6226 | 0.0772 | 73.1 |  | -17.8659 | 0.0904 | 82.2 | |
| MR1000 | -24.7723 | 0.0736 | 64.6 |  | -20.3682 | 0.0883 | 56.1 |  | -25.0353 | 0.0773 | 70.6 |  | -20.2738 | 0.0947 | 71.5 | |
| MR0100 | -24.7519 | 0.0735 | 64.6 |  | -20.3609 | 0.0883 | 55.3 |  | -25.0308 | 0.0773 | 70.5 |  | -20.2668 | 0.0948 | 71.8 | |
| MR0010 | 0.8791 | 0.0375 | 94.8 |  | 0.4286 | 0.0400 | 95.3 |  | 0.6967 | 0.0440 | 93.6 |  | 0.6053 | 0.0544 | 93.7 | |
| MR0001 | -24.6733 | 0.0733 | 64.9 |  | -20.2862 | 0.0880 | 56.4 |  | -24.9447 | 0.0771 | 70.7 |  | -20.1677 | 0.0943 | 71.2 | |
| MR1100 | -24.7528 | 0.0735 | 64.7 |  | -20.3663 | 0.0883 | 55.3 |  | -25.0318 | 0.0773 | 70.6 |  | -20.2743 | 0.0948 | 71.8 | |
| MR1010 | 0.9792 | 0.0378 | 94.4 |  | 0.3517 | 0.0400 | 95.5 |  | 0.7704 | 0.0443 | 94.0 |  | 0.4767 | 0.0547 | 94.0 | |
| MR1001 | -24.7595 | 0.0735 | 64.4 |  | -20.3603 | 0.0883 | 56.3 |  | -25.0251 | 0.0773 | 70.7 |  | -20.2708 | 0.0947 | 71.5 | |
| MR0110 | 0.9981 | 0.0378 | 94.6 |  | 0.3992 | 0.0401 | 95.4 |  | 0.7992 | 0.0442 | 94.3 |  | 0.5221 | 0.0548 | 94.1 | |
| MR0101 | -24.7411 | 0.0735 | 64.6 |  | -20.3554 | 0.0883 | 55.6 |  | -25.0174 | 0.0773 | 70.6 |  | -20.252 | 0.0947 | 71.7 | |
| MR0011 | 0.8138 | 0.0378 | 94.4 |  | 0.3335 | 0.0398 | 95.5 |  | 0.5850 | 0.0443 | 94.3 |  | 0.4763 | 0.0545 | 93.9 | |
| MR1110 | 1.0119 | 0.0378 | 94.5 |  | 0.3659 | 0.0400 | 95.6 |  | 0.8044 | 0.0443 | 94.1 |  | 0.4838 | 0.0547 | 94.1 | |
| MR1101 | -24.7426 | 0.0735 | 64.7 |  | -20.3577 | 0.0883 | 55.3 |  | -25.0223 | 0.0773 | 70.5 |  | -20.2694 | 0.0948 | 71.6 | |
| MR1011 | 1.0156 | 0.0379 | 94.5 |  | 0.2986 | 0.0400 | 95.8 |  | 0.8112 | 0.0445 | 94.3 |  | 0.4145 | 0.0546 | 94.2 | |
| MR0111 | 0.9284 | 0.0379 | 94.5 |  | 0.2969 | 0.0400 | 95.6 |  | 0.7086 | 0.0445 | 94.0 |  | 0.4110 | 0.0546 | 94.1 | |
| MR1111 | 1.0442 | 0.0380 | 94.3 |  | 0.3080 | 0.0400 | 95.7 |  | 0.8376 | 0.0446 | 94.0 |  | 0.4168 | 0.0547 | 94.3 | |

The true values of $\Delta\left( t=10 \right)$ or $\Delta\left( t=20 \right)$ are 0.2524 and 0.3816, respectively

Bias (%): mean relative bias; RMSE: root mean square error; CR: coverage rate; IPW: inverse probability weighting; OR: outcome regression; MR, multiply robust. MR estimators are denoted as “MR-0000”, where each digit of the four numbers, from left to right, indicates if $\pi^{1}\left( \boldsymbol{X};\boldsymbol{\beta}^{1} \right)$, $\pi^{2}\left( \boldsymbol{X};\boldsymbol{\beta}^{2} \right)$, $m^{1}\left( \boldsymbol{X,}Z;\boldsymbol{\gamma}^{1} \right)$ or $m^{2}\left( \boldsymbol{X,}Z;\boldsymbol{\gamma}^{2} \right)$ is included in the estimator (“1” means yes and “0” means no).

**Table S5.** The triple-negative breast cancer data analysis: estimated $\Delta\left( t^{*} \right)$for $t^{*}=1, 2, 3, 4, 5$and 6 years using different methods

|  | $\hat{\Delta}_{\mathrm{mr}}\left( t^{*}=1 \right)$ (95% CI) | $\hat{\Delta}_{\mathrm{mr}}\left( t^{*}=2 \right)$ (95% CI) | $\hat{\Delta}_{\mathrm{mr}}\left( t^{*}=3 \right)$ (95% CI) | $\hat{\Delta}_{\mathrm{mr}}\left( t^{*}=4 \right)$ (95% CI) | $\hat{\Delta}_{\mathrm{mr}}\left( t^{*}=5 \right)$ (95% CI) | $\hat{\Delta}_{\mathrm{mr}}\left( t^{*}=6 \right)$ (95% CI) |
| --- | --- | --- | --- | --- | --- | --- |
| IPW.model1 | 0.0484(0.0311-0.0657) | 0.0373(0.0164-0.0582) | 0.0294(0.0070-0.0519) | 0.0325(0.0090-0.0560) | 0.0275(0.0012-0.0538) | 0.0355(0.0042-0.0667) |
| IPW.model2 | 0.0481(0.0306-0.0656) | 0.0375(0.0163-0.0586) | 0.0299(0.0073-0.0525) | 0.0327(0.0091-0.0563) | 0.0281(0.0015-0.0548) | 0.0356(0.0043-0.0670) |
| OR.model1 | 0.0415(0.0236-0.0595) | 0.0342(0.0175-0.0509) | 0.0286(0.0111-0.0462) | 0.0371(0.0175-0.0567) | 0.0315(0.0109-0.0521) | 0.0434(0.0167-0.0701) |
| OR.model2 | 0.0414(0.0235-0.0592) | 0.0342(0.0175-0.0509) | 0.0286(0.0111-0.0461) | 0.0371(0.0175-0.0567) | 0.0315(0.0109-0.0521) | 0.0435(0.0167-0.0703) |
| MR1000 | 0.0582(0.0366-0.0798) | 0.0481(0.0232-0.0731) | 0.0406(0.0147-0.0666) | 0.0418(0.0150-0.0686) | 0.0375(0.0075-0.0676) | 0.0453(0.0106-0.0800) |
| MR0100 | 0.0581(0.0360-0.0802) | 0.0484(0.0232-0.0736) | 0.0413(0.0151-0.0674) | 0.0422(0.0153-0.0690) | 0.0386(0.0082-0.0690) | 0.0459(0.0111-0.0806) |
| MR0010 | 0.0330(0.0169-0.0492) | 0.0256(0.0099-0.0414) | 0.0271(0.0089-0.0453) | 0.0375(0.0168-0.0583) | 0.0320(0.0105-0.0535) | 0.0437(0.0171-0.0704) |
| MR0001 | 0.0329(0.0168-0.0490) | 0.0256(0.0099-0.0414) | 0.0271(0.0089-0.0453) | 0.0375(0.0167-0.0582) | 0.0320(0.0104-0.0535) | 0.0438(0.0170-0.0705) |
| MR1100 | 0.0581(0.0361-0.0802) | 0.0484(0.0233-0.0736) | 0.0413(0.0151-0.0674) | 0.0422(0.0153-0.0690) | 0.0385(0.0082-0.0689) | 0.0458(0.0111-0.0805) |
| MR1010 | 0.0467(0.0283-0.0651) | 0.0384(0.0177-0.0591) | 0.0342(0.0118-0.0565) | 0.0369(0.0130-0.0609) | 0.0336(0.0059-0.0613) | 0.0421(0.0099-0.0743) |
| MR1001 | 0.0468(0.0282-0.0654) | 0.0384(0.0177-0.0592) | 0.0343(0.0119-0.0567) | 0.0370(0.0130-0.0610) | 0.0338(0.0061-0.0615) | 0.0422(0.0099-0.0744) |
| MR0110 | 0.0468(0.0277-0.0659) | 0.0385(0.0176-0.0594) | 0.0345(0.0122-0.0569) | 0.0369(0.0131-0.0607) | 0.0342(0.0064-0.0620) | 0.0423(0.0103-0.0744) |
| MR0101 | 0.0470(0.0276-0.0664) | 0.0386(0.0176-0.0596) | 0.0346(0.0122-0.0570) | 0.0370(0.0131-0.0609) | 0.0343(0.0065-0.0622) | 0.0424(0.0103-0.0744) |
| MR0011 | 0.0325(0.0165-0.0484) | 0.0256(0.0098-0.0414) | 0.0270(0.0087-0.0453) | 0.0373(0.0165-0.0582) | 0.0317(0.0100-0.0534) | 0.0434(0.0166-0.0702) |
| MR1110 | 0.0468(0.0278-0.0659) | 0.0385(0.0176-0.0594) | 0.0345(0.0121-0.0568) | 0.0369(0.0130-0.0607) | 0.0342(0.0063-0.0620) | 0.0423(0.0102-0.0744) |
| MR1101 | 0.0470(0.0277-0.0663) | 0.0386(0.0176-0.0596) | 0.0346(0.0121-0.0570) | 0.0370(0.0131-0.0609) | 0.0343(0.0064-0.0621) | 0.0424(0.0102-0.0745) |
| MR1011 | 0.0456(0.0283-0.0629) | 0.0380(0.0176-0.0584) | 0.0336(0.0115-0.0557) | 0.0366(0.0128-0.0603) | 0.0336(0.0059-0.0613) | 0.0422(0.0099-0.0744) |
| MR0111 | 0.0455(0.0277-0.0633) | 0.0378(0.0172-0.0585) | 0.0337(0.0116-0.0559) | 0.0363(0.0126-0.0600) | 0.0341(0.0061-0.0621) | 0.0423(0.0101-0.0745) |
| MR1111 | 0.0455(0.0278-0.0633) | 0.0379(0.0173-0.0586) | 0.0337(0.0116-0.0559) | 0.0364(0.0127-0.0601) | 0.0340(0.0061-0.0620) | 0.0423(0.0101-0.0745) |

IPW: inverse probability weighting; OR: outcome regression; CI, confidence interval; MR, multiply robust; MR estimators are denoted as “MR-0000”, where each digit of the four numbers, from left to right, indicates if $\pi^{1}\left( \boldsymbol{X};\boldsymbol{\beta}^{1} \right)$, $\pi^{2}\left( \boldsymbol{X};\boldsymbol{\beta}^{2} \right)$, $m^{1}\left( \boldsymbol{X},Z;\boldsymbol{\gamma}^{1} \right)$ or $m^{2}\left( \boldsymbol{X},Z;\boldsymbol{\gamma}^{2} \right)$ is included in the estimator (“1” means yes and “0” means no).


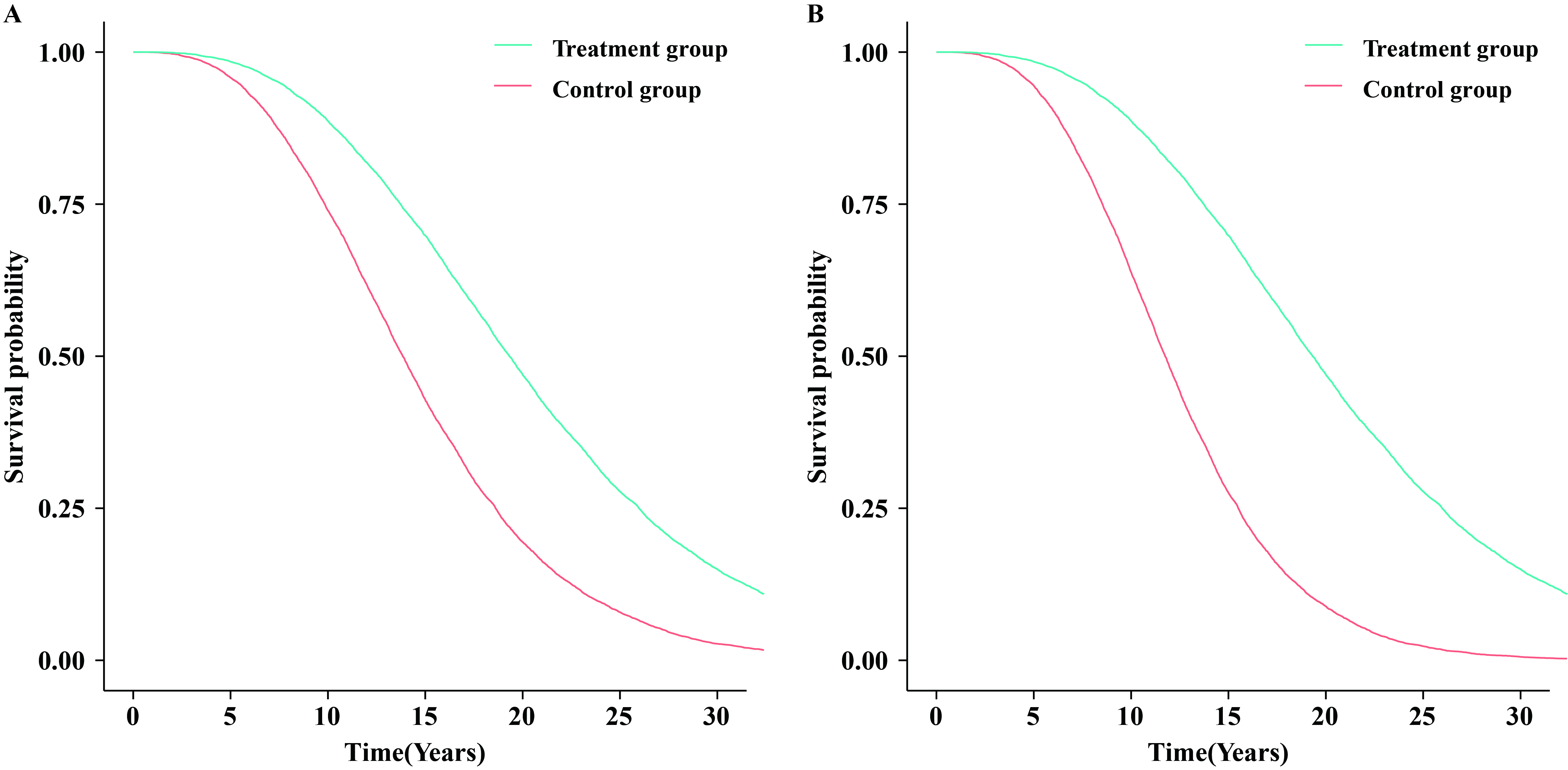


Figure S1 Weibull survival functions defining simulation scenario. Figure A shows proportional hazards assumption holds; and Figure B shows that proportional hazards assumption dose not hold
